# Identification of Key Targets of Herbal Compounds for Liver Fibrosis Using Network Pharmacology Combined With Transcriptomics

**DOI:** 10.1155/grp/9985154

**Published:** 2026-04-14

**Authors:** Chen Chen, Bo Huang, Mengting Hu, Shengpeng Yang, Bo Lan, Dejun Cui

**Affiliations:** ^1^ Department of Gastroenterology, Guizhou Provincial People’s Hospital, Guiyang, Guizhou Province, China, 5055.cn; ^2^ Guizhou Medical University, Guiyang, China, gmc.edu.cn; ^3^ Department of Hepatobiliary Surgery, Guizhou Provincial People’s Hospital, Guiyang, Guizhou Province, China, 5055.cn; ^4^ Department of Pharmacy, Guizhou Provincial People’s Hospital, Guiyang, Guizhou Province, China, 5055.cn

**Keywords:** biomarkers, Chinese herbal compound prescription, liver fibrosis, nomogram, single-cell RNA sequencing

## Abstract

Chinese herbal compound prescriptions have demonstrated efficacy in preventing and treating liver fibrosis (LF), though their mechanisms remained unclear. This study is aimed at identifying diagnostic biomarkers and elucidating the molecular mechanism underlying the effects of the TCM prescription on LF. LF‐related datasets (GSE162694, GSE84044, and GSE136103) were obtained from a public database. Active ingredient–related target genes (AIRTGs) and LF‐related target genes (LFRTGs) were intersected with differentially expressed genes (DEGs) between the LF and normal control (NC) group to select candidate genes. Subsequently, biomarkers for LF diagnosis were determined using Boruta and LASSO algorithms, receiver operating characteristic (ROC), and expression analyses. A nomogram was constructed to evaluate the capability of these biomarkers for predicting LF risk. Furthermore, GSEA and immunoinfiltration analysis were conducted, along with single‐cell analysis to identify relevant cell types in LF. COL3A1 and ALOX5 were identified as diagnostic biomarkers for LF, and the nomogram was proven effective in predicting LF risk. GSEA showed that COL3A1 might play vital roles in cell growth and differentiation, extracellular matrix organization, and cell–matrix interactions. The functions of ALOX5 might be associated with cell–cell interaction, cytoskeletal regulation, and so forth. Immunoinfiltration analysis revealed that activated dendritic cells (DCs) were highly infiltrated, whereas monocytes were less infiltrated in LF. COL3A1 expression was positively correlated with monocytes, but both COL3A1 and ALOX5 showed negative correlations with activated DCs. Single‐cell analysis identified nine cell types, with macrophages, B cells, and mesenchyme cells emerging as key cell types. Cell communication analysis demonstrated stronger interactions between macrophages and mesenchymal cells in the LF group. Pseudotime analysis unveiled that the expression of ALOX5 was upregulated and then downregulated, whereas that of COL3A1 was gradually downregulated during the midstage and stabilized thereafter. COL3A1 and ALOX5 may serve as biomarkers for the diagnosis and treatment of LF with Chinese herbal compound prescriptions, contributing to more accurate diagnosis and improved LF therapy.

## 1. Introduction

Liver fibrosis (LF) is a critical pathological stage in chronic liver disease, marked by persistent liver injury and excessive deposition of extracellular matrix (ECM) components (1), such as proteoglycans, collagens, glycoproteins, fibronectin, and laminin (2, 3), leading to architectural disruption and loss of liver function (4). Without timely intervention, LF can progress to cirrhosis and liver failure (5). Current management strategies include mesenchymal stem cell therapy, bariatric surgery, lifestyle modifications, and pharmacological treatments; for example, resmetirom, as a THR‐*β* agonist, has reached Phase III trials for LF (6). However, no drug has yet been approved by the FDA or EMA specifically for LF treatment. While many antifibrotic candidates are shown to be effective in animal models, their performance in clinical trials remains limited (7). Thus, elucidating the molecular mechanism and identifying novel biomarkers are essential for developing effective treatment strategies for LF.

The pathogenesis of LF has been extensively studied, with activated hepatic stellate cells (HSCs) as a primary contributor to LF formation, reversal, and progression (2). In normal conditions, HSCs remain dormant and are primarily involved in vitamin A and fat storage, supporting liver metabolism and energy homeostasis (8). Upon injury, HSCs activate into myofibroblasts, expressing *α*‐smooth muscle actin (*α*‐SMA) and acquiring profibrogenic transcriptional and secretory profiles (9), which disrupt liver sinus balance and aggravate LF progression (10). Additionally, M1 macrophages promote HSC activation through proinflammatory factors such as TNF‐*α*, IL‐1*β*, and IL‐6, while M2 macrophages attenuate fibrosis via anti‐inflammatory cytokines and ECM degradation (11). Despite these insights, effective treatments for LF remain limited (12). Currently, antifibrotic strategies are aimed at inhibiting ECM production, promoting its breakdown, inducing apoptosis of activated HSCs, or suppressing HSC activation (13). Traditional Chinese medicine (TCM) prescription comprises multiple active components and has shown significant therapeutic potential in LF. For example, phillygenin (PHI) demonstrates hepatoprotective and anti‐inflammatory effects by inhibiting HSC activation (14). Fraxini Cortex extracted from *Fraxinus chinensis* Roxb exhibits (15) antioxidant, antibacterial, and anti‐inflammatory properties, contributing to its efficacy in liver and intestinal disorders (16). These findings underscored the value of TCM as a rich resource for exploring therapeutic targets. Network pharmacology (17) integrates omics, system biology, and computational biology methods (18) and is a useful tool to elucidate the mechanisms of multicomponent drugs of TCM prescriptions within disease networks (19).

The scRNA‐seq analysis uncovers the distribution and functional status of different cell types at the cellular level (20). This advanced technology greatly improves the resolution and accuracy of cell heterogeneity analysis, reveals dynamic changes in cell status during disease progression, and captures variations in therapeutic response of different individuals (21). Therefore, in this study, we integrated public scRNA‐seq and bulk RNA‐seq data with information on a Chinese herbal compound prescription (e.g., Radix Codonopsis and *Citrus reticulata* peel) for LF treatment to identify potential biomarkers and elucidate the underlying molecular mechanisms of the therapeutic effects of the prescription. Furthermore, we developed a nomogram incorporating these biomarkers to facilitate individualized diagnosis and investigated their involved pathways, immune infiltration patterns, and cell differentiation characteristics.

## 2. Materials and Methods

### 2.1. Data Gathering

The Gene Expression Omnibus (GEO, http://www.ncbi.nlm.nih.gov/geo/) database was accessed to obtain two transcriptomic datasets (GSE162694 and GSE84044) and a scRNA‐seq dataset (GSE136103) related to LF. The GSE162694 dataset (platform: GPL21290) comprised high‐throughput sequencing data from 112 biopsies of nonalcoholic fatty liver disease (NAFLD) patients with fibrosis in the liver tissue biopsies of normal subjects. Microarray sequencing data from unfibrotic (*N* = 43) and fibrotic (*N* = 81) hepatic tissue samples from 124 patients with chronic hepatitis B (CHB) in the GSE84044 dataset (platform: GPL570) were collected. In GSE136103 (platform: GPL20301), hepatic tissue samples from five healthy subjects and five patients with fibrosis in NAFLD were used for single‐cell analysis.

### 2.2. Screening of Candidate Genes

The DEGs (|Log2fold − change (FC)| > 0.5), *p*.adj < 0.05) between LF and normal control (NC) groups in GSE162694 dataset were identified using the R package “DESeq2” (Ver. 1.36.0) (22). Volcano plots were drawn by the R package “ggplot2” (Ver. 3.3.0) (23) to visualize the DEGs and highlight the Top 10 most significant genes. Expression patterns of DEGs were displayed in a heat map using the R package “Pheatmap” (Ver. 0.7.7) (24). The active compounds of Chinese herbal compound prescription (Radix Codonopsis, *Citrus reticulata* peel, *Atractylodes macrocephala*, *Platycodon grandiflorus* root, *Fritillaria thunbergii* Miq., *Boswellia serrata*, Ecliptae herba, Ephedrae herba, *Ganoderma lucidum*, and *Cuminum cyminum* L.) and active ingredient–related target genes (AIRTGs) were searched using the TCMSP database (https://old.tcmsp-e.com/tcmsp.php) (oral bioavailability [OB] ≥ 30*%* and drug likeness [DL] ≥ 0.18). LF‐related target genes (LFRTGs) were obtained from the intersection of LF‐associated targets in GeneCards (https://www.genecards.org/) and DisGeNET (https://www.disgenet.org/) databases. Subsequently, candidate genes involved in active ingredients of TCM prescriptions in LF were derived by intersecting DEGs, AIRTGs, and LFRTGs.

### 2.3. Functional Enrichment Analysis and Protein–Protein Interaction (PPI) Network Analysis

To elucidate the functional roles of candidate genes, Gene Ontology (GO) and Kyoto Encyclopedia of Genes and Genomes (KEGG) analysis was conducted employing the R package “clusterProfiler” (Ver. 3.8.1) (25), with an adj.*p* < 0.05 denoting statistical significance. A PPI network was developed using the STRING database (https://string-db.org, confidence = 0.4).

### 2.4. Acquisition of Biomarkers

Boruta algorithm in the R package “Boruta” (Ver. 8.0.0) (26, 27) was utilized to select candidate genes with a *Z* value higher than the maximum *Z* value of the shadow feature. These genes were considered Boruta‐featured genes with important properties. To reduce the feature dimension, the R package “glmnet” (Ver. 4.1‐4) (28) was applied to develop a LASSO model. Key features were selected at the optimal lambda value where model accuracy peaked, as determined by 10‐fold cross‐validation (family = binomial). The Spearman correlation analysis was then applied to assess interactions among these genes, and a correlation network was constructed. The diagnostic performance of each feature gene was evaluated using ROC curves generated with the “timeROC” package (v1.0.3) (29). Genes with AUC > 0.70 in both the GSE162694 and GSE84044 datasets were considered as candidate biomarkers. Differential expression of these candidates between the LF and NC groups was further validated using the Wilcoxon rank‐sum test in both datasets. Biomarkers for LF were ultimately identified based on consistent differential expression (*p* < 0.05) and uniform expression trends across the two datasets.

### 2.5. Establishment of a Nomogram

A nomogram model was established via the R package “rms” (Ver. 6.5‐0.0) (29) to comprehensively demonstrate the prediction ability of the biomarkers. The performance of the nomogram was reflected in the calibration curve, ROC curve, confusion matrix, decision curve, and clinical impact curve. Decision curves were plotted with the R package “DecisioPrimaryurve” (Ver. 6.5‐0.0).

### 2.6. GSEA and Biomarker‐Based Network Construction

KEGG pathways enriched by the biomarkers were identified through GSEA. Correlation coefficients between the biomarkers and other genes in the GSE162694 dataset were calculated to rank the genes. The R package “clusterProfiler” was then used to perform GSEA on the ranked genes (adj.*p* < 0.05). Using the miRTarBase (http://mirtarbase.cuhk.edu.cn/) database, microRNAs (miRNAs) targeting the biomarkers and their corresponding lncRNAs were predicted using the R package “MultiMiR” (Ver. 1.20.0) (30). Additionally, the GeneMANIA database (https://genemania.org/) was utilized to develop a gene–gene interaction (GGI) network to identify functionally similar genes and reveal their interactions with the biomarkers. Finally, a biomarker–active ingredient–Chinese herbal compound network was constructed to explore the interactions between active ingredients in TCM prescriptions for the treatment of LF and the biomarkers.

### 2.7. Immunoinfiltration Analysis

To investigate the roles of immune cells in LF, the CIBERSORT tool in the R package “immunedeconv” (Ver. 2.1.0) (31, 32) was employed to estimate the relative proportions of 22 immune cells in both the LF and NC groups in the GSE162694 dataset (immune cells without abundance in all samples were eliminated). Differences in the abundance of immune cells between the two groups were compared by the Wilcoxon rank‐sum test (*p* < 0.05), with significantly different cells classified as discrepant immune cells. The correlation between discrepant immune cells and the biomarkers was defined via the Spearman correlation analysis (*p* < 0.05).

### 2.8. Quality Control (QC), Dimensionality Reduction, and Cell Annotation

The “CreateSeuratObject” function in the “Seurat” package (Ver. 5.0.1) (33) was employed to preprocess data from the GSE136103 dataset for removing low‐quality cells. Cells with nFeature_RNA > 4000, nFeature_RNA < 200, nCount_RNA > 12,000, and a mitochondrial gene ratio exceeding 10% were excluded. The data were standardized to ensure comparability between different cells and different genes. The Top 1000 highly variable genes (HVGs) between cells were identified and normalized by “FindVariableFeatures” function (selection.method = vst, nfeatures = 1000) and “ScaleData” function, followed by performing principal component analysis (PCA) for dimensional reduction. The “JackStraw” and “ScoreJackStraw” functions were used for linear dimension reduction. The percentage of variance explained by each PC was visualized (*p* < 0.05). Furthermore, the UMAP algorithm was utilized for dimensionality reduction to obtain the cell clusters at a resolution ratio = 1 (34). Next, cell types were annotated based on the expression of marker genes from the CellMarker website (http://xteam.xbio.top/CellMarker/) and a referenced study (35).

### 2.9. Expression Analysis, Cell Communication, and Pseudotime Analyses

The average expression levels of the biomarkers in each cell type were estimated, and the distribution of gene expression in cell types was displayed. The R package “CellChat” (Ver. 1.6.1) (36) was employed to analyze receptor–ligand communication based on the LF and NC samples in the GSE136103 dataset. Subsequently, pseudotime analysis was conducted on the key cell types via the “Monocle” (Ver. 2.26.0) (37) package in R. Specifically, HVGs within the key cell types were extracted as feature genes. Dimensionality reduction and cell sequencing were conducted utilizing the DDRTree algorithm for the pseudotime analysis. Moreover, dynamic expression changes of the biomarkers during differentiation of cell types were observed.

### 2.10. Statistical Analysis

In the current study, the R software (Ver. 4.2.0) was used in computational analyses. The Wilcoxon rank‐sum test was employed to evaluate two‐group differences, with a *p* value < 0.05 considered statistically significant ( ^∗^ *p* < 0.05,  ^∗∗^ *p* < 0.01,  ^∗∗∗^ *p* < 0.001, and  ^∗∗∗∗^ *p* < 0.0001).

## 3. Results

### 3.1. Candidate Genes Might Play a Crucial Role in Regulating Immune Responses, Stress Reactions, and Cell Proliferation

A total of 2598 DEGs were identified between the LF and NC groups in the GSE162694 dataset. In particular, the LF group contained 2331 overexpressed and 267 downregulated DEGs (Figure 1a,b). A number of 227 AIRTGs were retrieved from TCMSP, and 2461 LFRTGs were obtained from the GeneCards and DisGeNET databases. A total of 38 candidate genes were in the intersections of the DEGs, AIRTGs, and LFRTGs (Figure 1c). GO analysis showed that the candidate genes were related to the response to lipopolysaccharide, bacteria, and oxidative stress and showed positive regulation of leukocyte migration and smooth muscle cell proliferation (Figure 1d). Moreover, these genes were enriched into AGE‐RAGE signaling pathway, TNF signaling pathway, IL‐17 signaling pathway, and other KEGG pathways (Figure 1e). These results showed that the candidate genes might play a crucial role in regulating immune responses, stress reactions, and cell proliferation. The PPI network exhibited 382 interaction pairs formed by 38 candidate genes, including COL3A1‐JUN, ALOX5‐MPO, and ALOX5‐IL1B (Figure 1f).

Figure 1Identifying candidate genes and function enrichment analysis. (a) The volcano map of differentially expressed gene (DEG) analysis between the liver fibrosis (LF) and normal groups. (b) The heat map of the DEG expression. (c) Venn plot of overlap genes among DEGs, active ingredient–related target genes (AIRTGs), and LF‐related target genes (LFRTGs). (d) The Gene Ontology (GO) function enrichment analysis of these candidate genes. (e) The Kyoto Encyclopedia of Genes and Genomes (KEGG) pathway enrichment analysis of candidate genes. (f) Protein–protein interaction (PPI) network of these candidate genes.

**Figure:**
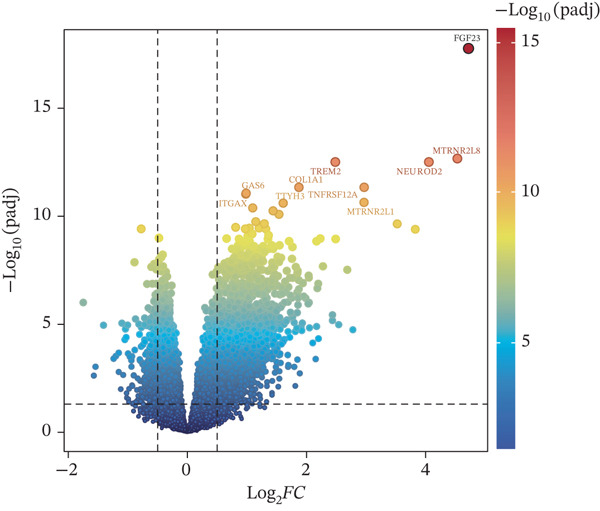
(a)

**Figure:**
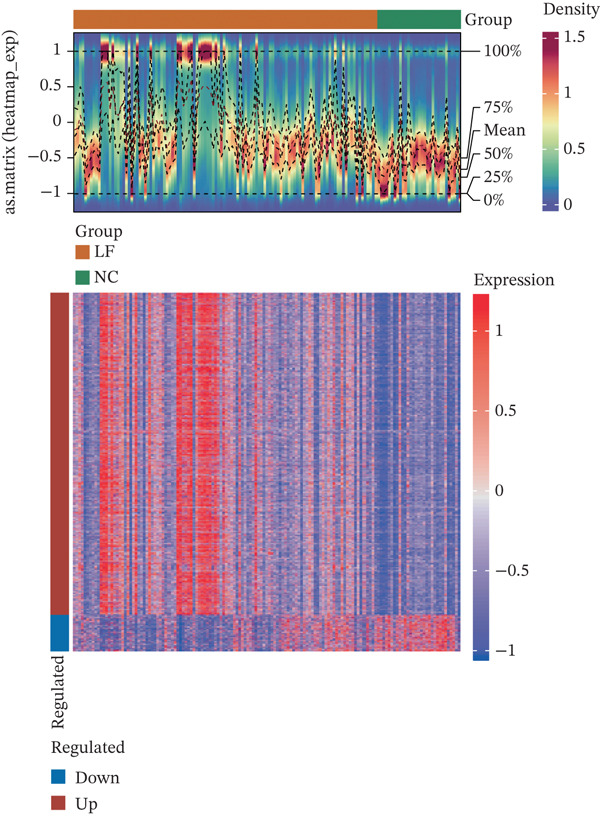
(b)

**Figure:**
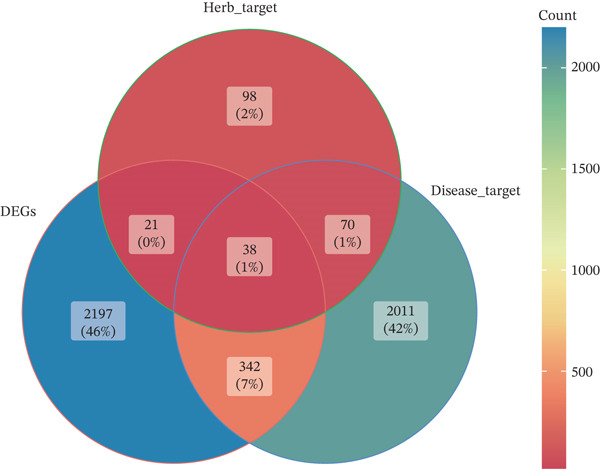
(c)

**Figure:**
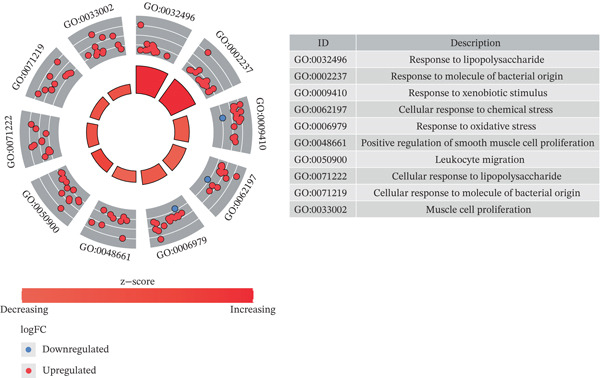
(d)

**Figure:**
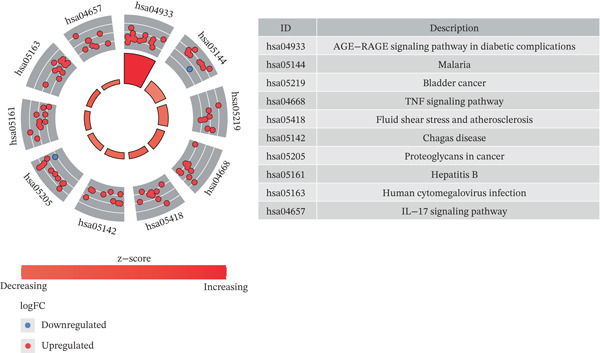
(e)

**Figure:**
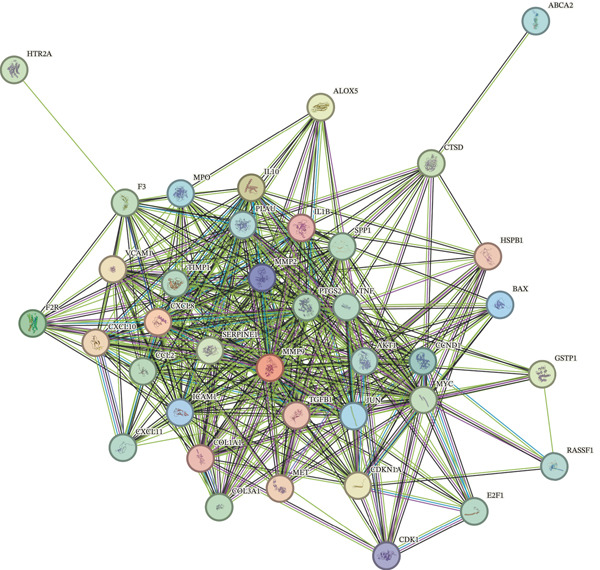
(f)

### 3.2. *COL3A1* and *ALOX5* Were Overexpressed Biomarkers for LF

A Boruta model was built using the 38 candidate genes, identifying 16 Boruta‐featured genes (TIMP1, TGFB1, SERPINE1, RASSF1, MYC, MMP2, MET, JUN, HTR2A, GSTP1, CTSD, COL3A1, COL1A1, CDKN1A, BAX, and ALOX5). Among these genes, COL3A1 had the highest importance score (Figure 2a,b). A sum of 12 LASSO feature genes (VCAM1, SERPINE1, RASSF1, MPO, MET, HTR2A, F2R, COL3A1, CDK1, BAX, ALOX5, and ABCA2) was obtained at the minimum lambda value of 0.01390364 (Figure 2c). A total of seven feature genes were selected by intersecting the genes obtained by the two algorithms (Figure S1A). The Spearman correlation analysis demonstrated that these seven feature genes formed close correlation pairs with each other. Specifically, COL3A1 showed positive correlations with ALOX5, BAX, and RASSF1 and negative correlations with HTR2A and MET. Additionally, ALOX5 was negatively correlated with HTR2A (Figure 2d). ROC analysis revealed that COL3A1 and ALOX5 achieved AUC values of 0.852 and 0.778, respectively, in the GSE162694 dataset and 0.82 and 0.80 in the GSE84044 dataset. Therefore, they were considered as candidate biomarkers in LF (Figure 2e and Figure S1B). Expression analysis further identified COL3A1 and ALOX5 as biomarkers for LF, based on their significant overexpression in the LF groups in both the GSE162694 and GSE84044 datasets (*p* < 0.001) (Figure 2f and Figure S1C).

Figure 2Determination of biomarker for diagnosis. (a) Boruta algorithm for screening Boruta‐featured genes. (b) The importance analysis of Boruta‐featured genes. (c) The LASSO algorithm for ascertaining the smallest lambda value of LASSO‐featured genes. (d) The correlation analysis among these feature genes. (e) Receiver operating characteristic (ROC) analysis of two biomarkers in GSE84044 dataset. (f) The expression difference of two biomarkers in the LF and control groups in GSE162694 database ( ^∗∗∗∗^ *p* < 0.0001).

**Figure:**
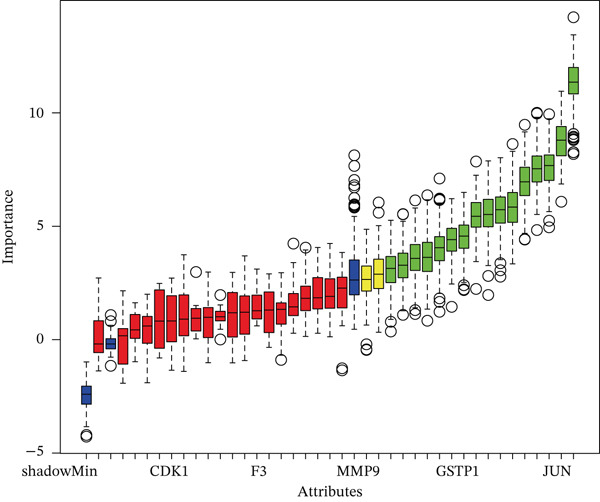
(a)

**Figure:**
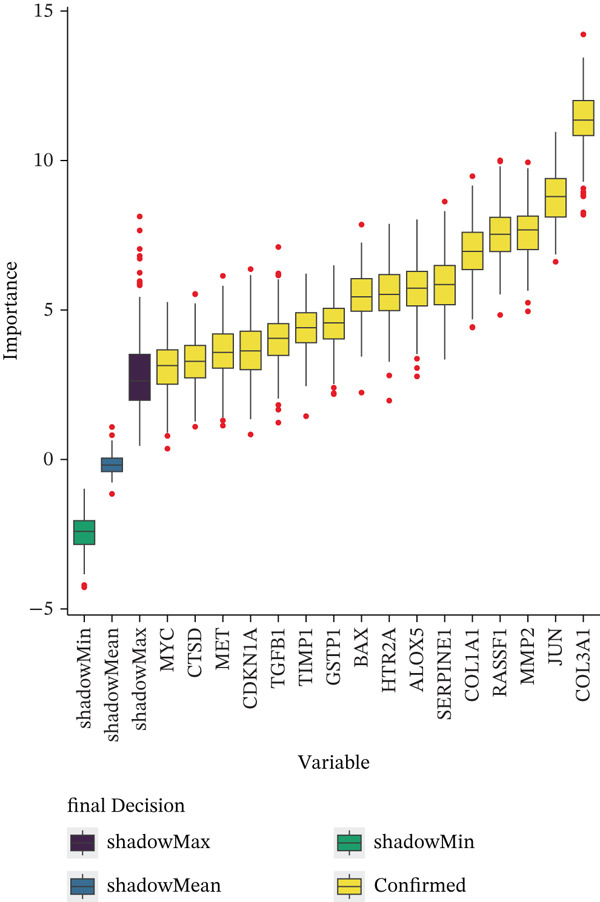
(b)

**Figure:**
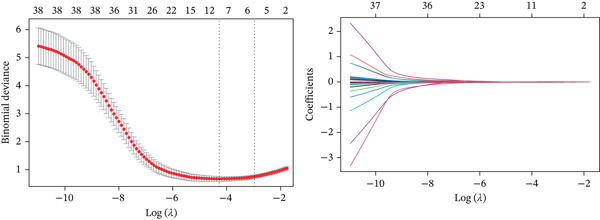
(c)

**Figure:**
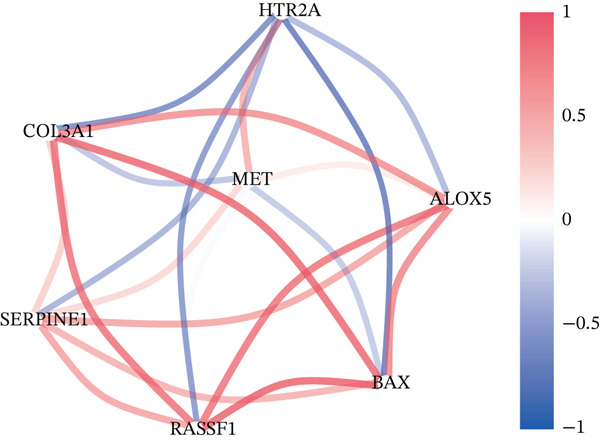
(d)

**Figure:**
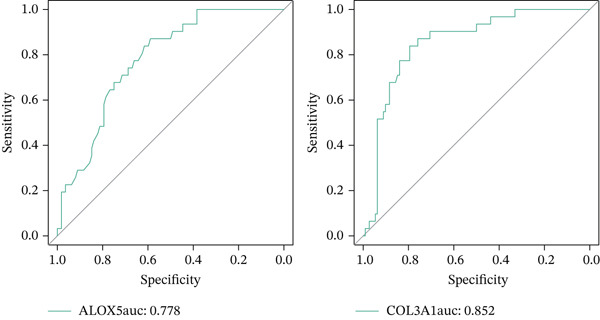
(e)

**Figure:**
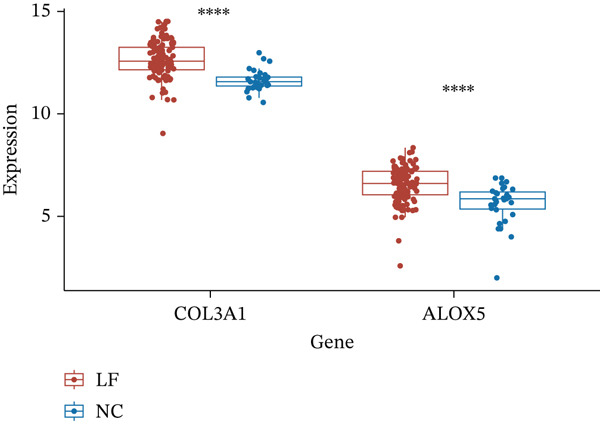
(f)

### 3.3. A Biomarker‐Based Nomogram Was an Effective Model for Predicting LF Risk

The nomogram model for predicting the probability of LF was built based on the expression of biomarkers. Obviously, the risk of LF increased with higher total points (Figure 3a). The nomogram demonstrated a high predictive accuracy, with a *C*‐index of 0.871 (Figure 3b) and an AUC value of 0.870, confirming its effectiveness (Figure 3c). The confusion matrix indicated an agreement rate of 82.031% between predicted and actual outcomes, reflecting high predictive ability (Figure 3d). The decision curve showed that the net benefit of the nomogram was the highest, further supporting its clinical utility (Figure 3e). According to the clinical impact curve, the prediction results of the nomogram model closely aligned with the actual outcomes once the threshold value exceeded 0.2 and were in complete agreement above 0.6, indicating excellent performance of the model (Figure 3f). In conclusion, the biomarker‐based nomogram was an effective model for predicting LF risk.

Figure 3A developing nomogram model and its features. (a) Construction of nomogram model. (b) The calibration curve of nomogram model. (c) ROC analysis of nomogram model. (d) The confusion matrix of predicted value of nomogram model. (e) The decision curve of nomogram model. (f) Clinical impact curve of nomogram model.

**Figure:**
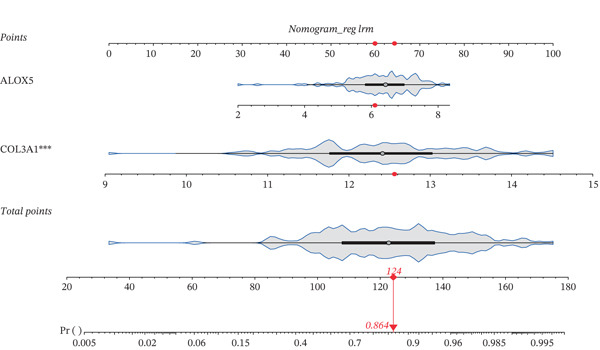
(a)

**Figure:**
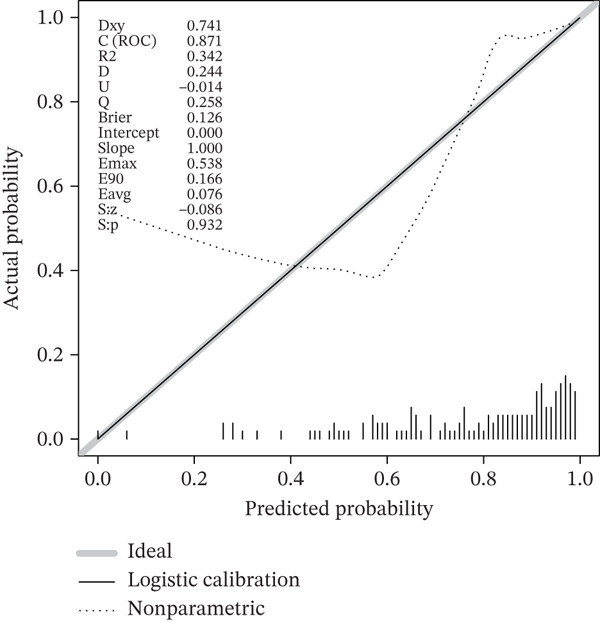
(b)

**Figure:**
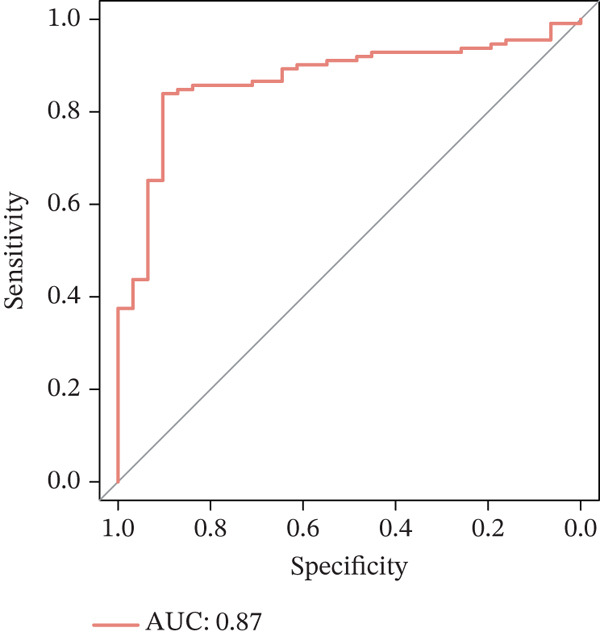
(c)

**Figure:**
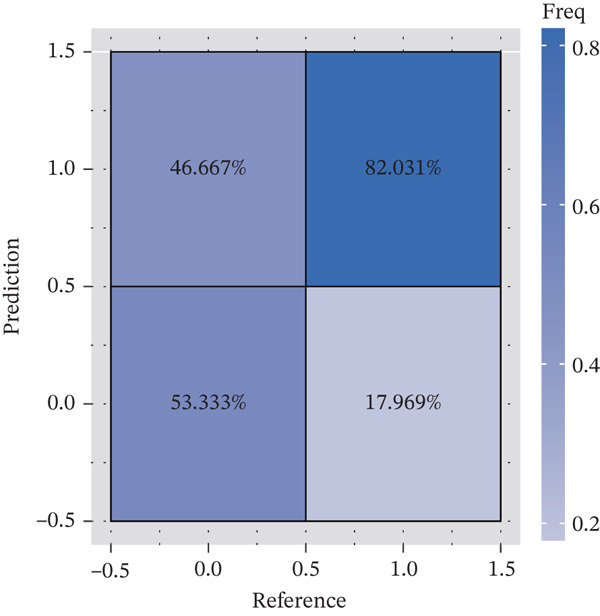
(d)

**Figure:**
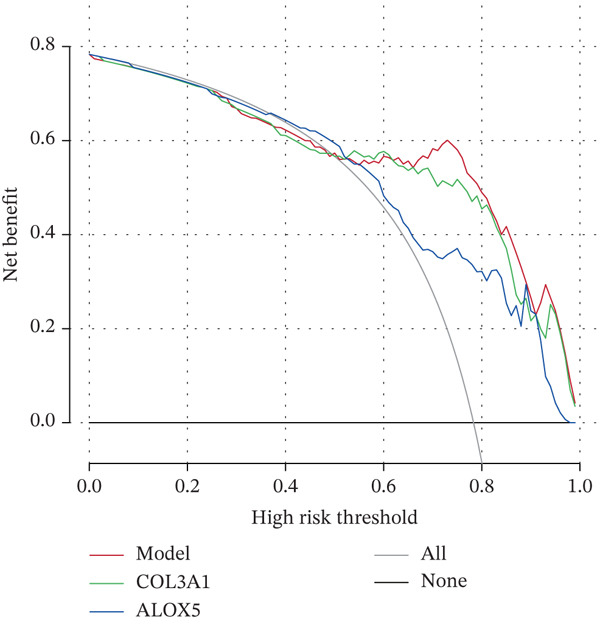
(e)

**Figure:**
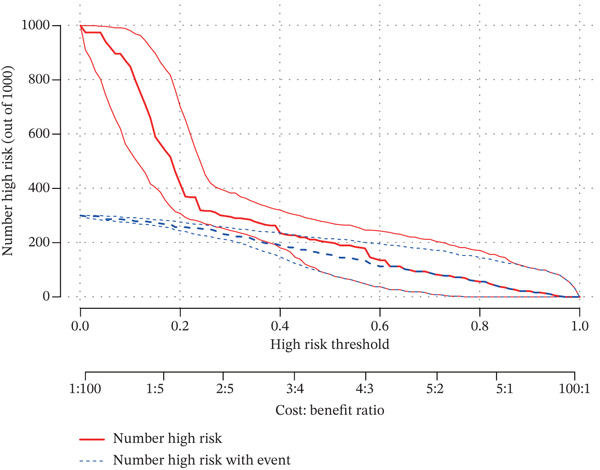
(f)

### 3.4. The Biomarker‐Related Functions Identified by GSEA

COL3A1 was significantly enriched in GO terms including platelet‐derived growth factor binding, ECM structural constituent, integrin binding, ECM organization, and ECM structure organization (Figure 4a). KEGG analysis showed that COL3A1 was enriched in focal adhesion, drug metabolism–cytochrome P450 pathways, and so forth (Figure 4b). These results suggested that COL3A1 might play vital roles in cell growth and differentiation, ECM organization, and cell–matrix interactions. ALOX5 was involved in positive regulation of leukocyte cell–cell adhesion, negative regulation of defense response, and negative regulation of immune response in GO and phagosome, phospholipase D signaling pathway, and regulation of actin cytoskeleton in KEGG (Figure 4c,d). In brief, the functions of ALOX5 might be associated with cell signaling, cell–cell interaction, cytoskeletal regulation, and immune response.

Figure 4Function enrichment analysis and network construction of two biomarkers. (a) GO term of the COL3A1 enrichment. (b) KEGG enrichment analysis of COL3A1. (c) GO term of the ALOX5 enrichment. (d) KEGG enrichment analysis of ALOX5. (e) The mRNA–miRNA–lncRNA network of two biomarkers. (f) Gene–gene interaction (GGI) network of two biomarkers. (g) Biomarker–active ingredient–Chinese herbal compound network of two biomarkers.

**Figure:**
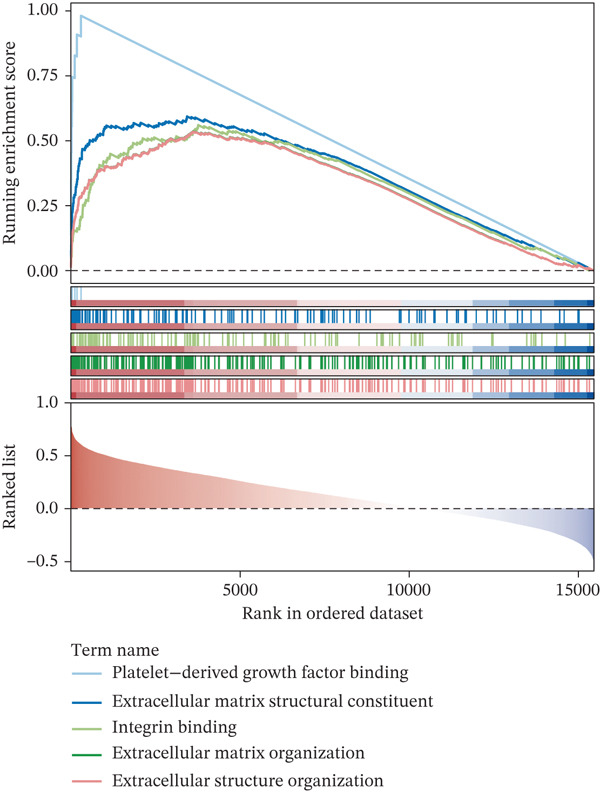
(a)

**Figure:**
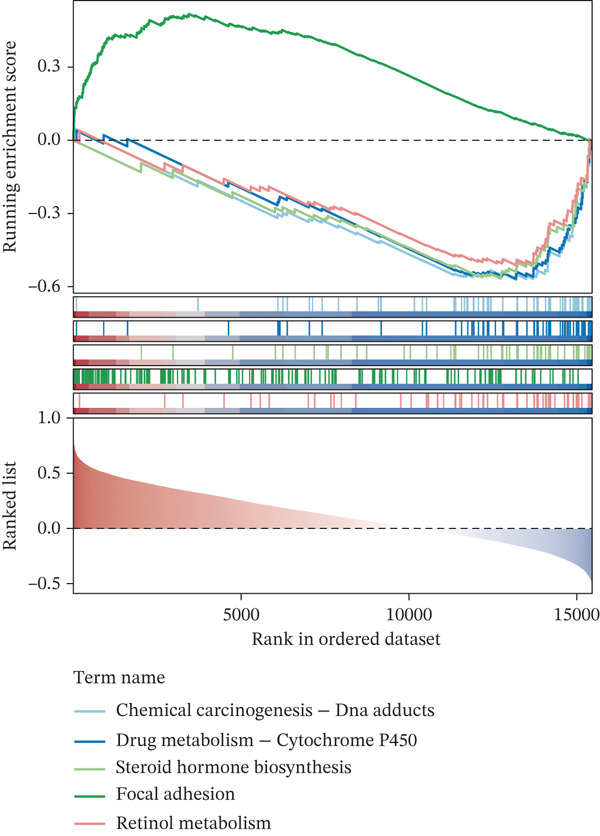
(b)

**Figure:**
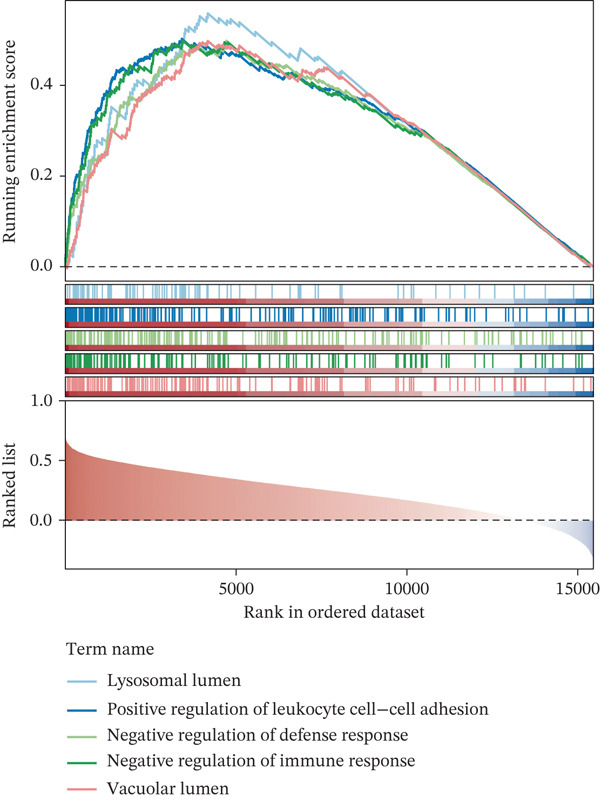
(c)

**Figure:**
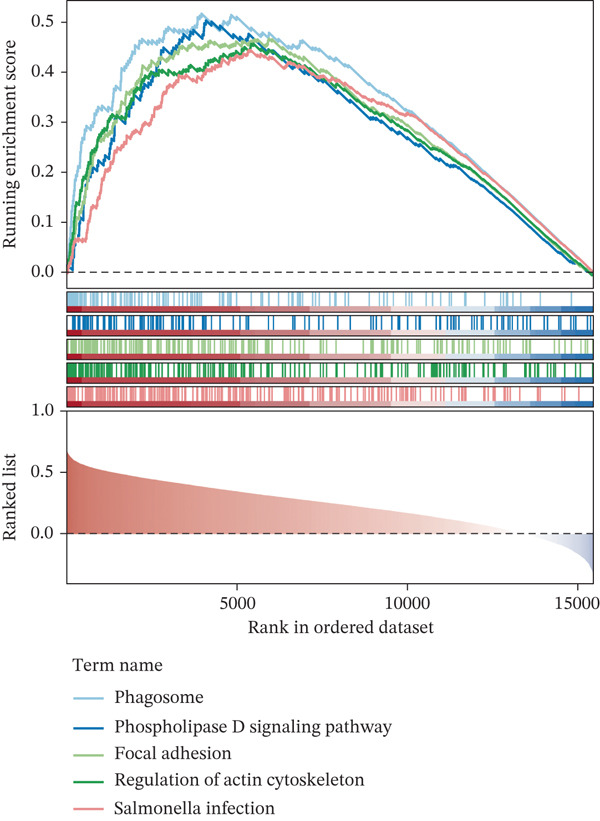
(d)

**Figure:**
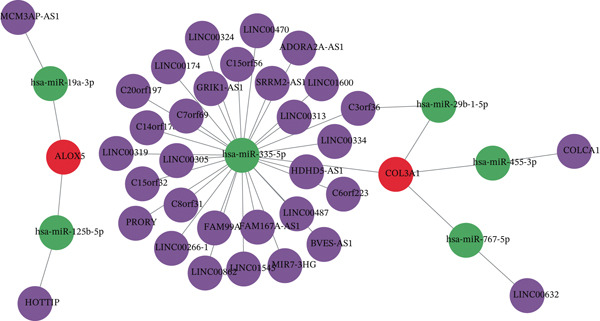
(e)

**Figure:**
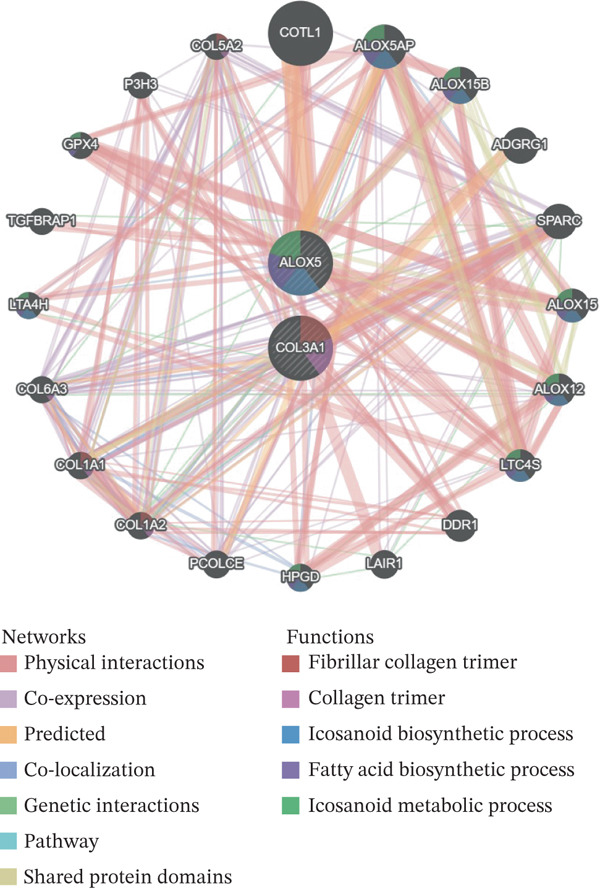
(f)

**Figure:**
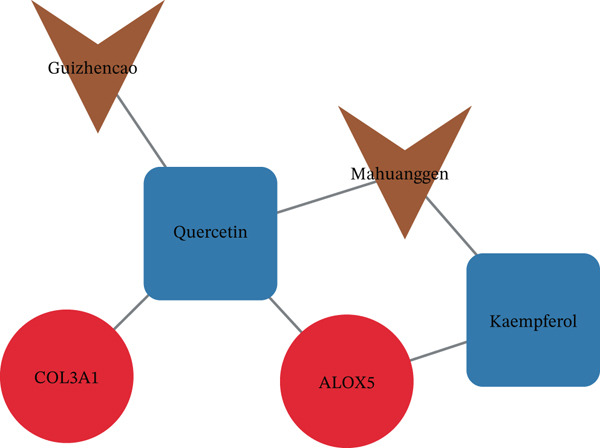
(g)

### 3.5. The Regulatory Network and Herbal Compound Interactions of the Biomarkers

The mRNA–miRNA–lncRNA network revealed that MCM3AP‐AS1 and HOTTIP might regulate the expression of ALOX5 via hsa‐miR‐19a‐3p and hsa‐miR‐125b‐5p, respectively. The expression of COL3A1 was directly regulated by hsa‐miR‐335‐5p, hsa‐miR‐29b‐1‐5p, hsa‐miR‐455‐3p, and hsa‐miR‐767‐5p (Figure 4e). A total of 20 genes with similar biological functions to the biomarkers were identified by the GeneMANIA database. As presented in the GGI network, ALOX5 was closely linked to ALOX5AP through shared protein domains and physical interactions. Similarly, COL3A1 and PCOLCE were correlated (Figure 4f). In the biomarker–active ingredient–Chinese herbal compound network, the biomarkers were linked to two active ingredients (quercetin and kaempferol) and two Chinese herbal compounds (*Boswellia serrata* [Gui Zhencao] and Ecliptae herba [Ma Huanggen]). Specifically, ALOX5 might target Ecliptae herba through quercetin and kaempferol (Figure 4g).

### 3.6. *COL3A1* Was Positively Correlated With Monocytes

The relative proportions of 21 immune cells in each LF and NC sample of the GSE162694 dataset were exhibited in a heat map (Figure 5a). Activated DCs were highly infiltrated, whereas monocytes showed low infiltration in LF samples (Figure 5b). COL3A1 was positively correlated with monocytes (cor = 0.32, *p* < 0.001), while COL3A1 (cor = −0.38, *p* < 0.001) and ALOX5 (cor = −0.43, *p* < 0.001) had a negative correlation with activated DCs (Figure 5c).

Figure 5Immune infiltration analysis. (a) The heat map of immune cell proportion in various samples. (b) The immune infiltration difference of immune cells among LF and normal groups. (c) The correlation between two biomarkers and differential immune cells.

**Figure:**
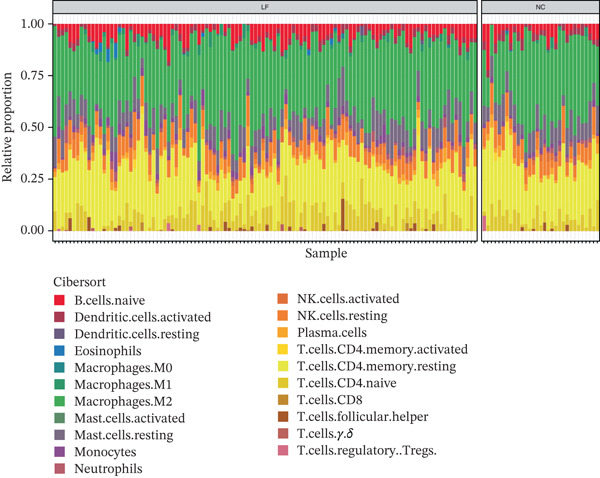
(a)

**Figure:**
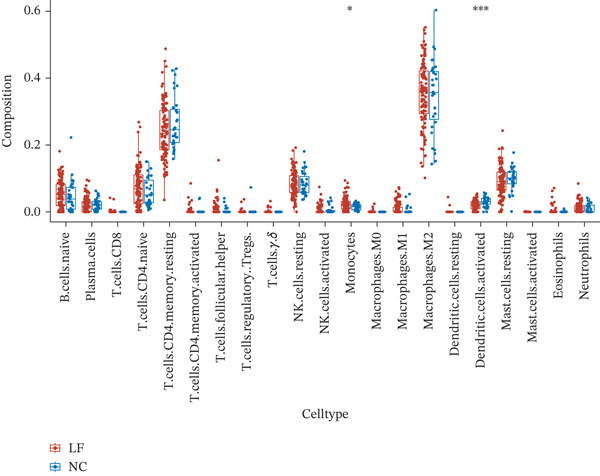
(b)

**Figure:**
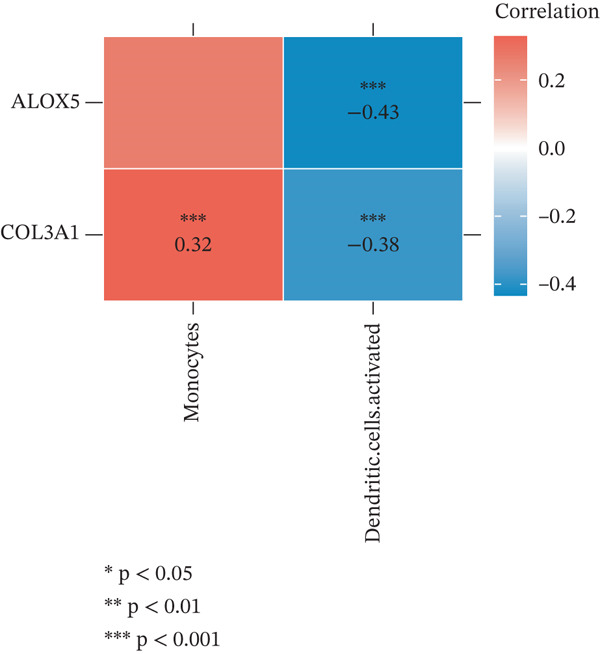
(c)

### 3.7. Macrophages, B Cells, and Mesenchyme Cells Were Identified as Key Cell Types

A total of 61,900 cells in the GSE136103 dataset were reduced to 55,473 cells after QC. Quality metrics including nFeature_RNA, nCount_RNA, and mitochondrial gene percentage before and after QC are shown in Figure S2A,B. The data variables were extracted and analyzed, revealing a well‐balanced distribution across the samples. No outlier samples were identified, indicating that there was no need for sample exclusion (Figure S2C). And the distribution of gene expression frequency after standardization is shown in Figure S2D. The red spots in Figure S2E represented the Top 1000 HVGs. PCA revealed clear clustering among samples without outliers (Figure S2F). The first 17 PCs were selected for dimensionality reduction and clustering, resulting in 23 cell clusters in the GSE13610 dataset (*p* < 0.05) (Figure S2G,H). These clusters were merged into nine cell types based on the expression of the marker genes in cell clusters (T cells, NK cells, macrophages, endothelial cells, epithelial cells, B cells, mesenchyme cells, cycling cells, and plasmacytoid DCs [pDCs]) (Figures 6a, 6b, and 6c). Next, we found that ALOX5 and COL3A1 had the highest expressions in macrophages and mesenchyme cells, respectively (Figure 6d). Cell communication analysis indicated stronger interaction between B cells and macrophages in the NC group compared to the LF group, whereas the interaction between macrophages and mesenchyme cells was stronger in the LF group (Figure 6e,f). Additionally, in both the NC group and LF group, MIF‐(CD74 + CD44) was a common signaling pathway mediating intercellular communication (Figure 6g,h). Given the correlation between the ALOX5 gene and monocytes (precursor cells of macrophages) and a high expression of the ALOX5 gene in macrophages, pseudotime analysis was performed on macrophages. HVGs within the macrophages were displayed in (Figure S2I). Analysis revealed that macrophages differentiated from Stage 1 (Figure 6i). During differentiation, ALOX5 expression initially increased and then decreased, whereas COL3A1 expression gradually declined in the middle stage and stabilized in the later stage (Figure 6j,k).

Figure 6Single‐cell RNA‐seq analysis. (a) The Uniform Manifold Approximation and Projection (UMAP) plot of mainly cell clusters. (b) The bubble plot of marker gene expression of cell clusters. (c) The bubble plot of marker gene expression of cell subtypes. (d) The expression of two biomarkers in specific cell types. (e) Cell communication analysis in the LF group. (f) Cell communication analysis in the control group. (g) The ligand–receptor interaction types in the LF groups. (h) The ligand–receptor interaction types in the normal groups. (i) Trajectory of macrophage development. (j) The expression of two biomarkers with pseudotime. (k) The heat map of two biomarker expressions with pseudotime.

**Figure:**
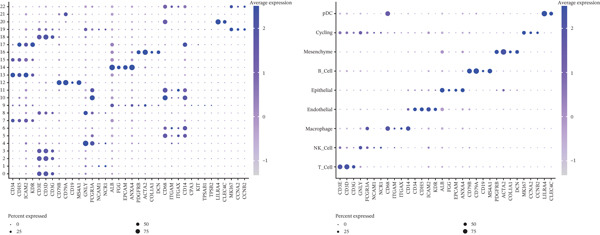
(a)

**Figure:**
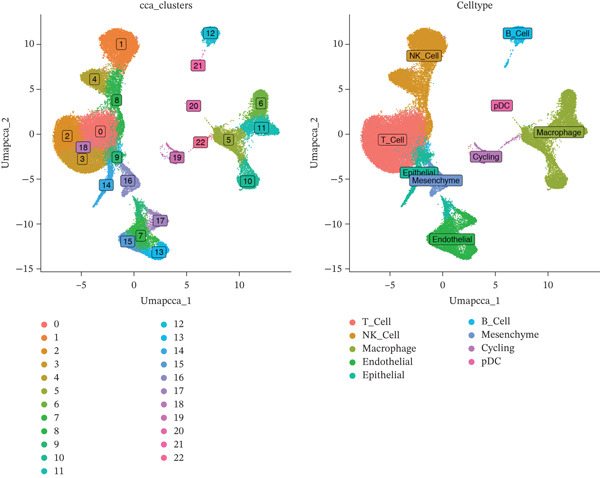
(b)

**Figure:**
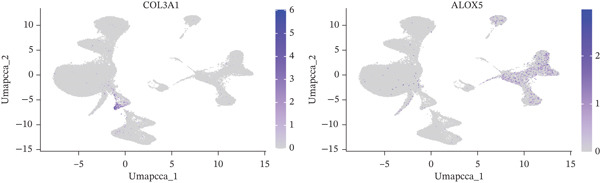
(c)

**Figure:**
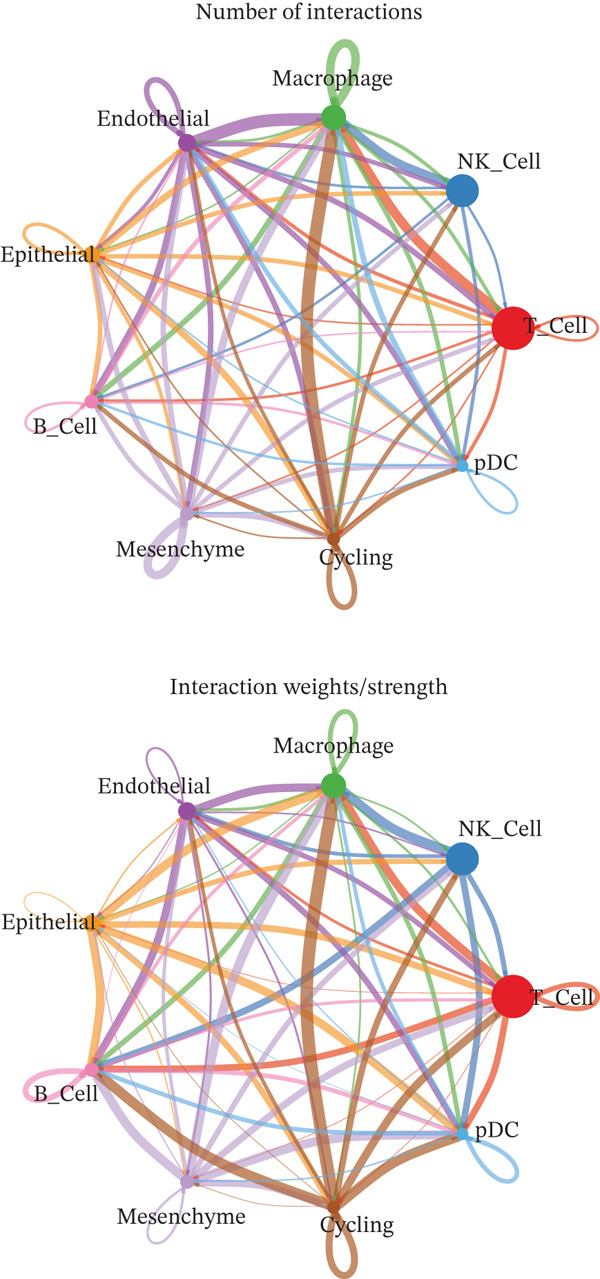
(d)

**Figure:**
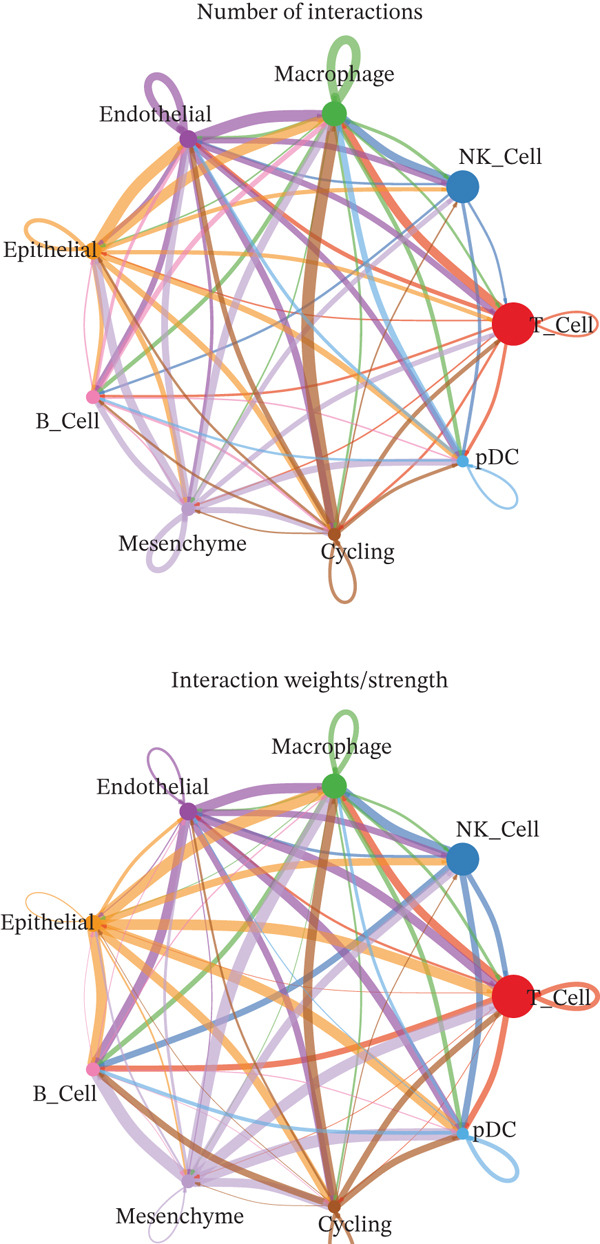
(e)

**Figure:**
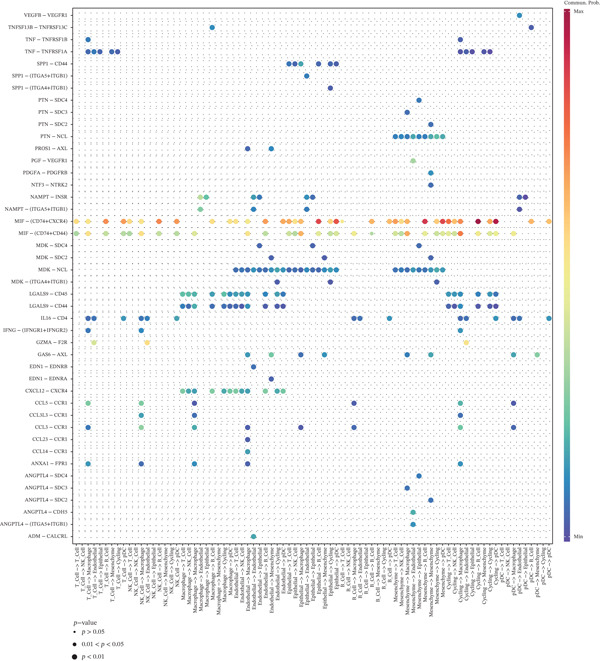
(f)

**Figure:**
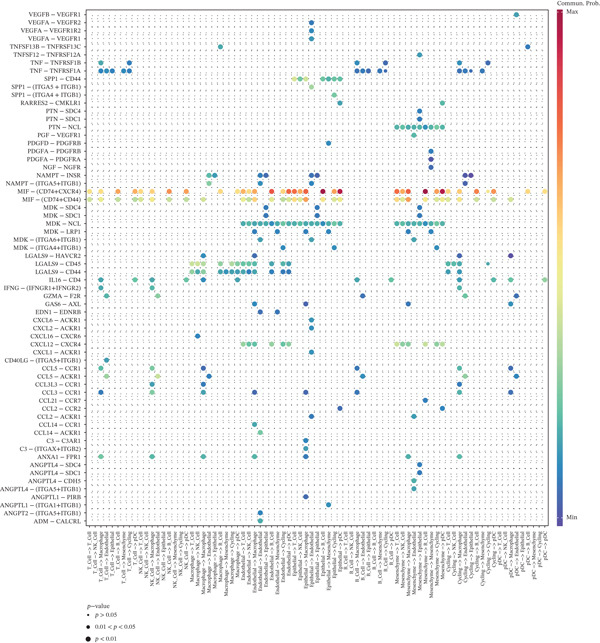
(g)

**Figure:**
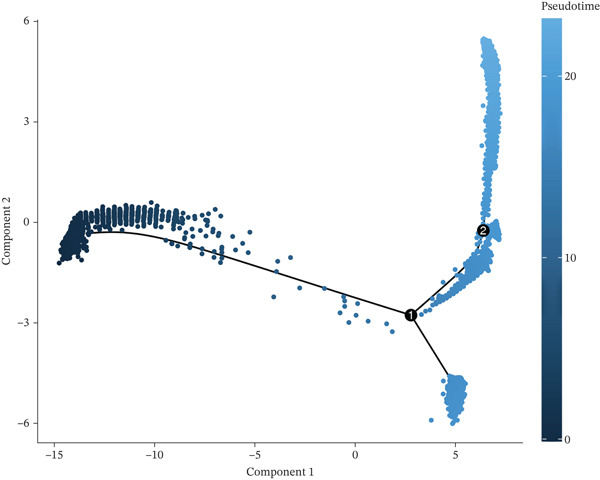
(h)

**Figure:**
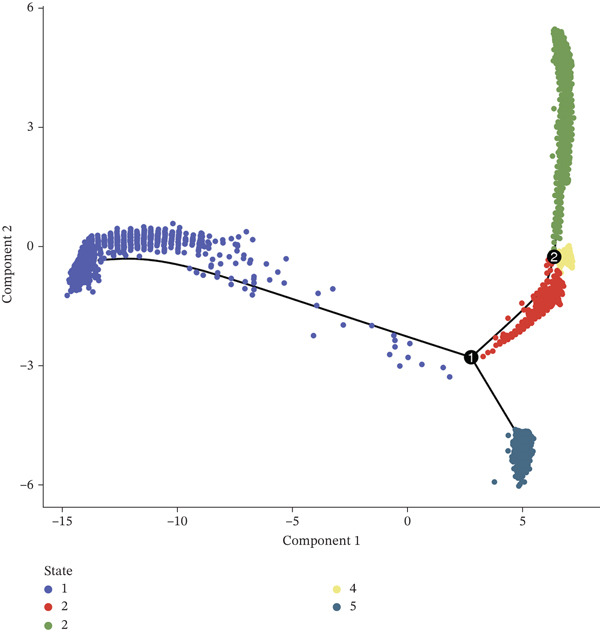
(i)

**Figure:**
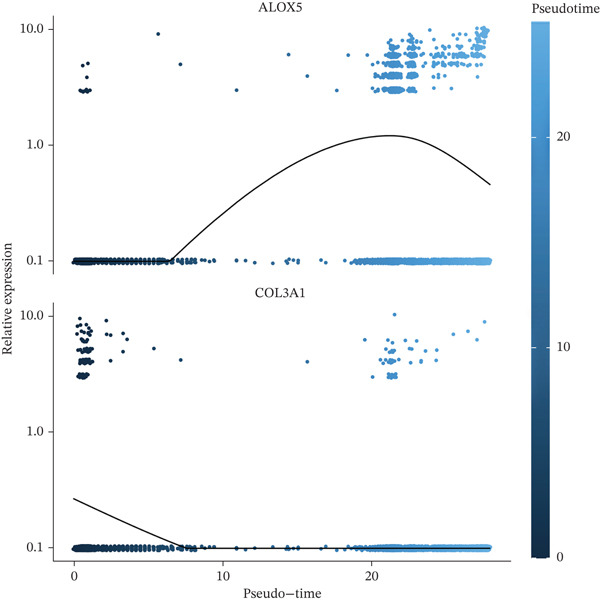
(j)

**Figure:**
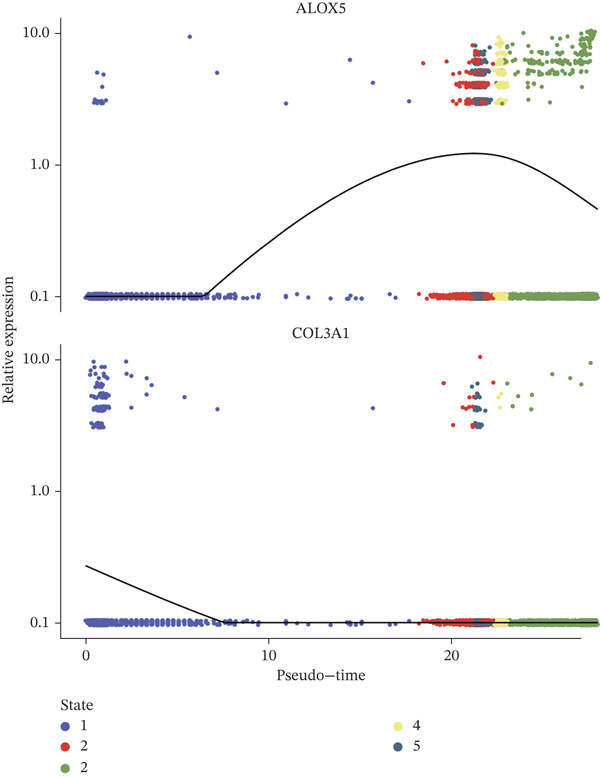
(k)

**Figure:**
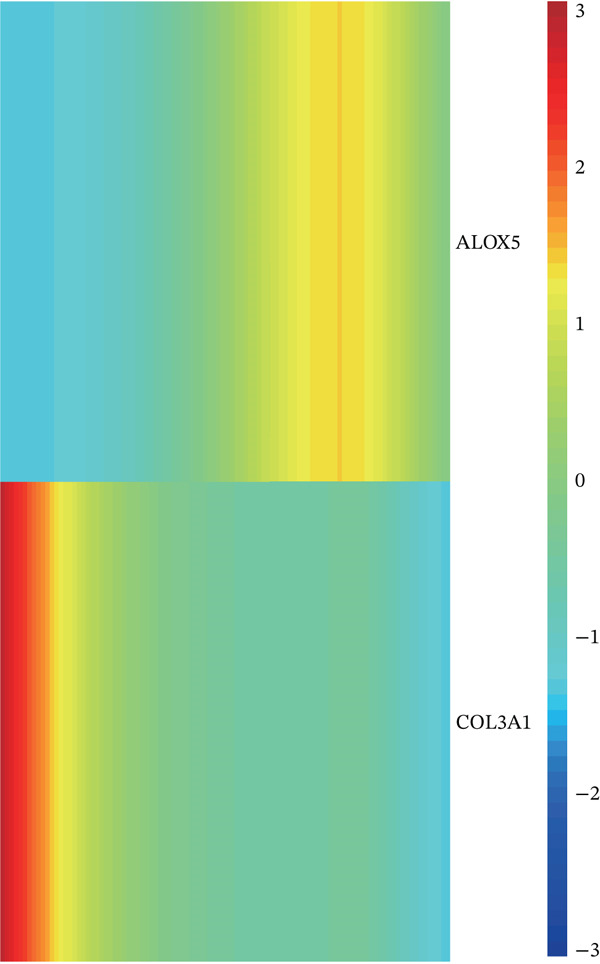
(l)

## 4. Discussion

LF is a progressive pathological process resulting from chronic liver injury, characterized by excessive ECM deposition mainly produced by activated HSCs (38). These cells upregulate *α*‐SMA, Collagen I (COL1), and various fibrogenic factors, ultimately leading to structural and functional impairment of the liver (12). Although MSC‐based therapies have shown promise in modulating ECM remodeling, their precise regulatory mechanisms remain unclear, highlighting an urgent need for innovative treatment strategies (39, 40). TCM prescriptions, with multitarget and anti‐inflammatory properties, provide an attractive therapeutic option (41). In this study, we combined classical TCM prescriptions with computational analyses to identify potential biomarkers and elucidate therapeutic mechanisms for LF. Importantly, scRNA‐seq analysis has proven effective in resolving disease heterogeneity and revealing new targets in complex pathologies such as drug‐resistant cancers (42). Building on this concept, we applied scRNA‐seq analysis to reveal specific expression patterns of distinct cell types and their role in driving fibrosis progression. These findings underscored the utility of single‐cell approaches in biomarker discovery, providing new opportunities for the precise diagnosis and treatment of LF.

In this study, two biomarkers, COL3A1 and ALOX5, were selected by differential expression analysis, machine learning, and expression validation. Collagen alpha‐1(III) chain is an ECM protein (Type III collagen) encoded by the COL3A1 gene, which is the main structural component of cavity organs such as bowel, blood vessels, and uterus (43). Moreover, the Type III collagen is also a crucial wound healing signaling molecule that interacts with platelets in the blood clotting cascade (44). High level of COL3A1 in the ECM aggravates LF progression (45). Lv et al. reported that arctigenin alleviates CCl4‐induced LF through inhibiting *α*‐SMA and COL3A1 expression for HSC quiescence (46). ALOX5 encodes a lipoxygenase that catalyzes leukotriene production, contributing to inflammation and bronchoconstriction. Knocking out ALOX5 attenuates LF and inflammatory responses in Apoe‐/‐ mice (47), while its pharmacological inhibition ameliorates CCL4‐mediated liver damage (48). This evidence suggested that the two genes were involved in the pathogenesis of LF and represented potential therapeutic targets for LF.

miRNAs are short, single‐stranded endogenous RNAs, approximately 20 nucleotides in length. miRNAs negatively regulate gene expression by inhibiting translation or shearing of target genes (49), affecting apoptosis, immune response, embryogenesis, and cancerogenesis (50). This study investigated the miRNA network of the two biomarkers. Previous studies reported that hsa‐miR‐19a‐3p targets TGF‐*β* RII mRNA to inhibit the autophagy of human cardiac fibroblasts (HCFs) (51). Overexpression of miR‐125b‐5p supports M1 macrophage polarization and its exosomes function (52). Hsa‐miR‐133a‐3p targeting COL1A1 in HSCs negatively regulates LF (53), and hsa‐miR‐29a‐3p targets COL3A1 to inhibit fibroblast‐derived COL1 production (54). Collectively, these results demonstrated that miRNAs regulate ECM via various mechanisms (55). Our findings further revealed novel miRNA–mRNA interactions in LF, including hsa‐miR‐19a‐3p and hsa‐miR‐125b‐5p for ALOX5, as well as hsa‐miR‐335‐5p and hsa‐miR‐29b‐1‐5p for COL3A1, highlighting their potential roles in fibrotic regulation.

Quercetin and kaempferol, both flavonoid compounds with antioxidative and anti‐inflammatory properties, were identified as active ingredients targeting the two biomarkers. Quercetin has been reported to exert anti‐LF effect by inhibiting the anti‐inflammation signaling pathways of NF‐кB/IкB*α* and p38 MAPK to reduce the levels of *α*‐SMA, desmin, Colla‐1 and ‐2, TNF‐*α*, and IL‐6 and ‐1*β* (56). Another study reported that quercetin alleviates LPS‐induced myocardial injury through downregulating ALOX5 (57). Xu et al. demonstrated that kaempferol injection significantly decreases the collagen deposition and necroinflammatory scores of liver tissue through targeting collagen synthesis (COL1 and *α*‐SMA) and HSC activation in vivo and in vitro (58), suggesting that Ecliptae herba containing quercetin and kaempferol are the key herbs in this prescription.

In addition, LF progression involves the conjoint regulation of multiple cell types, such as DCs and macrophages (59). Activated DCs are professional antigen‐presenting cells that capture and process antigens and secrete diverse cytokines to initiate immune response. Under normal conditions, liver DCs are tolerogenic through inducing Tregs and reducing active T cells (60). However, upon liver injury, DCs obtain the capacity to activate HSCs, T cells, and NK cells for inflammation (61). Our results showed that the high infiltration of activated DCs may aggravate LF progression, while COL3A1 and ALOX5 were negatively correlated with activated DCs, suggesting a potential negative regulatory relationship between the expressions of the two biomarkers and DC infiltration. Macrophages have high heterogeneity and plasticity in many diseases, including LF (62). M1 macrophages increase the expression of proinflammatory TNF‐*α*, IL‐6, and IL‐1*β*, and their inflammatory property affects HSC activation (63). The scRNA‐seq analysis revealed high ALOX5 expression in macrophages, consistent with ALOX5 being predominantly expressed in macrophages (64) and involved in producing proinflammatory leukotrienes (65). Thus, high‐expressed ALOX5 may promote a proinflammatory M1 phenotype, accelerating LF progression. In summary, we employed an innovative method integrating network pharmacology and RNA‐seq analysis to discover two biomarkers for LF diagnosis, offering novel insight into the mechanism of TCM prescription for LF intervention. However, there were still some limitations. For example, the two biomarkers exhibited excellent diagnostic efficiency in the validation set, but their predictive accuracy required further verification in a clinical trial. In addition, the mechanism or molecular pathways of the biomarkers in affecting the LF progression remained unclear.

## 5. Conclusion

In this study, we identified two biomarkers, COL3A1 and ALOX5, using RNA‐seq and TCM prescription‐based analysis. The two biomarkers were all overexpressed in the LF samples, and potential novel miRNAs regulating their expression in LF progression have been identified. Our study may promote the understanding of the mechanism of TCM prescription in intervening the development of LF.

**Nomenclature**
LF: liver fibrosis
AIRTGs: active ingredient–related target genes
LFRTGs: LF‐related target genes
DEGs: differentially expressed genes
NC: normal control
LASSO: least absolute shrinkage and selection operator
GEO: Gene Expression Omnibus
ROC: receiver operating characteristic
GO: Gene Ontology
KEGG: Kyoto Encyclopedia of Genes and Genomes
AUC: area under curve
BP: biological process
MF: molecular function
CC: cellular component
DL: drug likeness
OB: oral bioavailability
DCs: dendritic cells
GSEA: gene set enrichment analysis

## Author Contributions

C.C.: conceptualization, data curation, validation, visualization, writing—original draft, and writing—review and editing. B.H.: data curation, validation, visualization, and writing—review and editing. M.H.: validation and writing—review and editing. S.Y.: visualization and writing—review and editing. B.L.: visualization and writing—review and editing. D.C.: conceptualization, supervision, and writing—review and editing.

## Funding

This research was funded by Cultivation Fund of National Natural Science Foundation (Grant No. qiankehe2018‐5764‐11), Guizhou Provincial Department of Science and Technology 2025 Basic Research Plan Youth Guidance Project Qiankehe Foundation (QN [2025] 287), and the Scientific Research Project of Guizhou Provincial Health Commission (gzwkj2022‐052).

## Disclosure

All authors read and approved the final manuscript.

## Conflicts of Interest

The authors declare no conflicts of interest.

## Supporting Information

Additional supporting information can be found online in the Supporting Information section.

## Supporting information


**Supporting Information 1** Figure S1. Overlap analysis, ROC curve analysis, and expression validation. (A) Venn diagram of overlapping genes identified by Boruta and LASSO algorithms. (B) ROC curve analysis in the GSE84044 dataset. (C) Expression validation in the GSE84044 dataset.

**Supporting Information 2** Figure S2. The quality control (QC) for single‐cell RNA‐seq analysis. (A) The nFeature, nCount, and mitochondria proportion detection before QC. (B) The nFeature, nCount, and mitochondria proportion detection after QC. (C) Outlier detection in samples. (D) The gene distribution frequency after standardization. (E) The volcano plot for Top 1000 HVG. (F) PCA analysis of cell clusters. (G) Regression analysis for the optimal number of clusters. (H) Scree plot for dimensionality reduction clustering. (I) The identifying HVGs of macrophages.

## Data Availability

The datasets analyzed for this study can be found in the Gene Expression Omnibus database (http://www.ncbi.nlm.nih.gov/geo) with the accession numbers GSE162694, GSE84044, and GSE136103.
