# Supplementary figures and images for: Identification of Key Targets of Herbal Compounds for Liver Fibrosis Using Network Pharmacology Combined With Transcriptomics
Source: Gastroenterol Res Pract. 2026 Apr 14;2026:9985154. doi: 10.1155/grp/9985154 (PMC13080265; doi:10.1155/grp/9985154)

**(A)**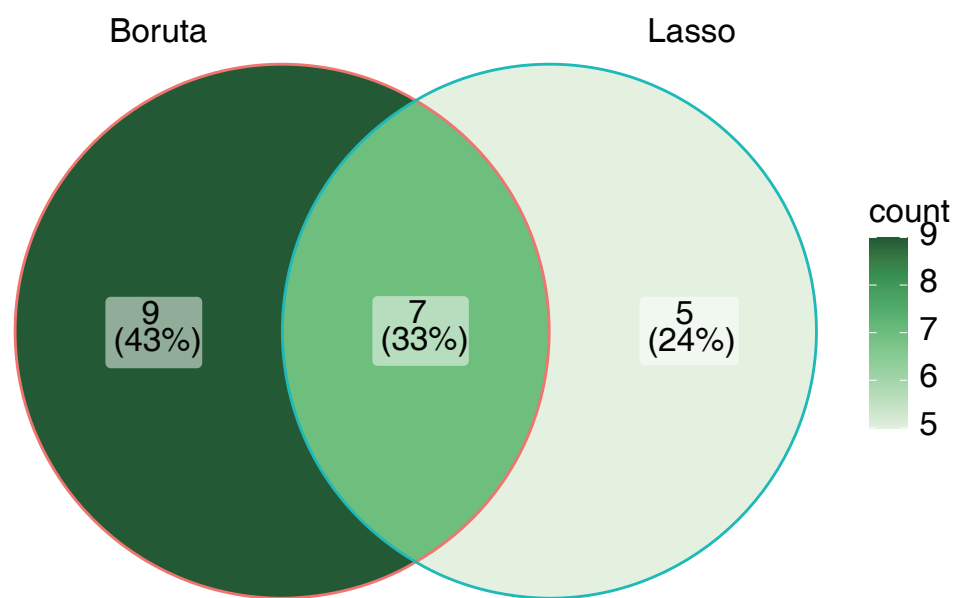**(B)**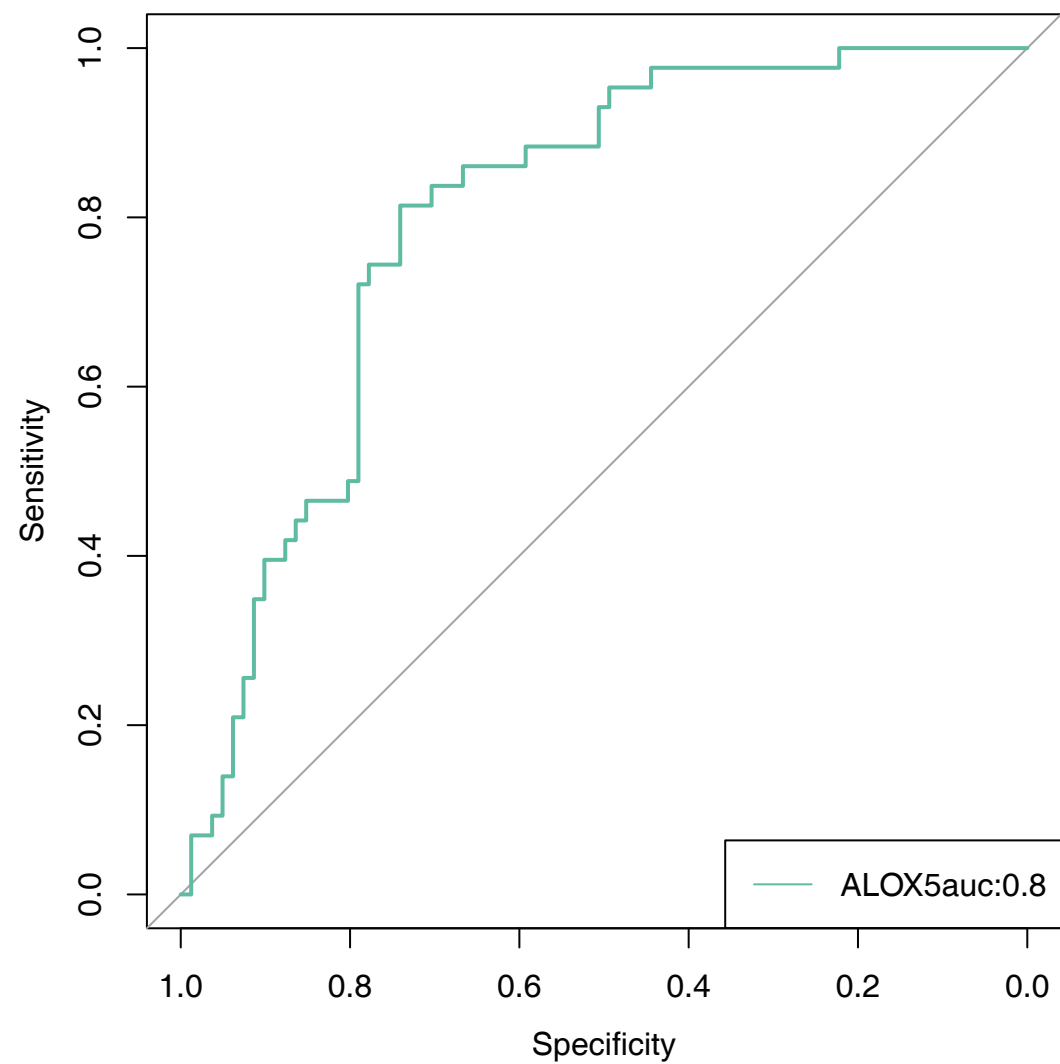**(C)**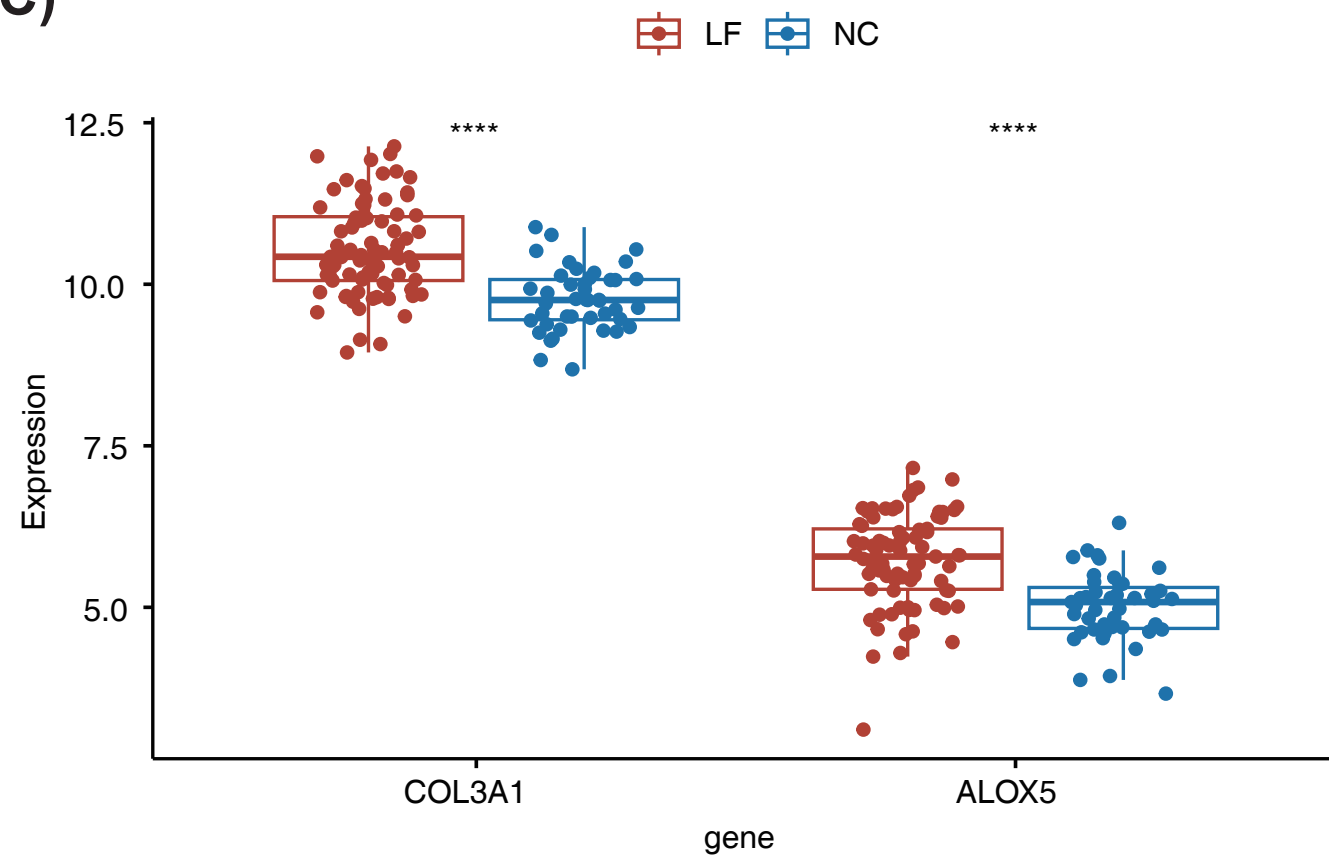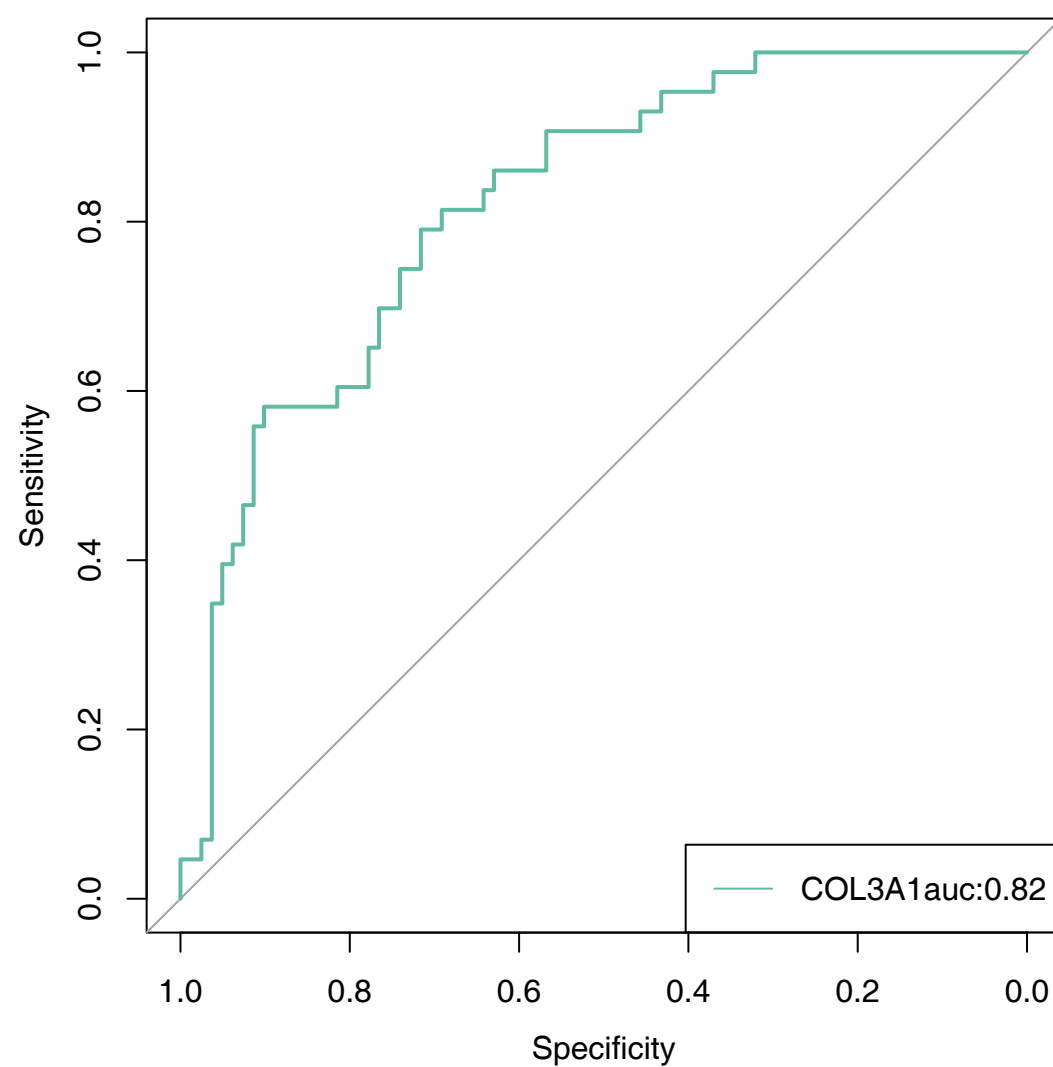

Supplement: Supplementary file 1 — Supporting Information 1 Figure S1. Overlap analysis, ROC curve analysis, and expression validation. (A) Venn diagram of overlapping genes identified by Boruta and LASSO algorithms. (B) ROC curve analysis in the GSE84044 dataset. (C) Expression validation in the GSE84044 dataset. [file GRP-2026-9985154-s002.pdf]

**(A)**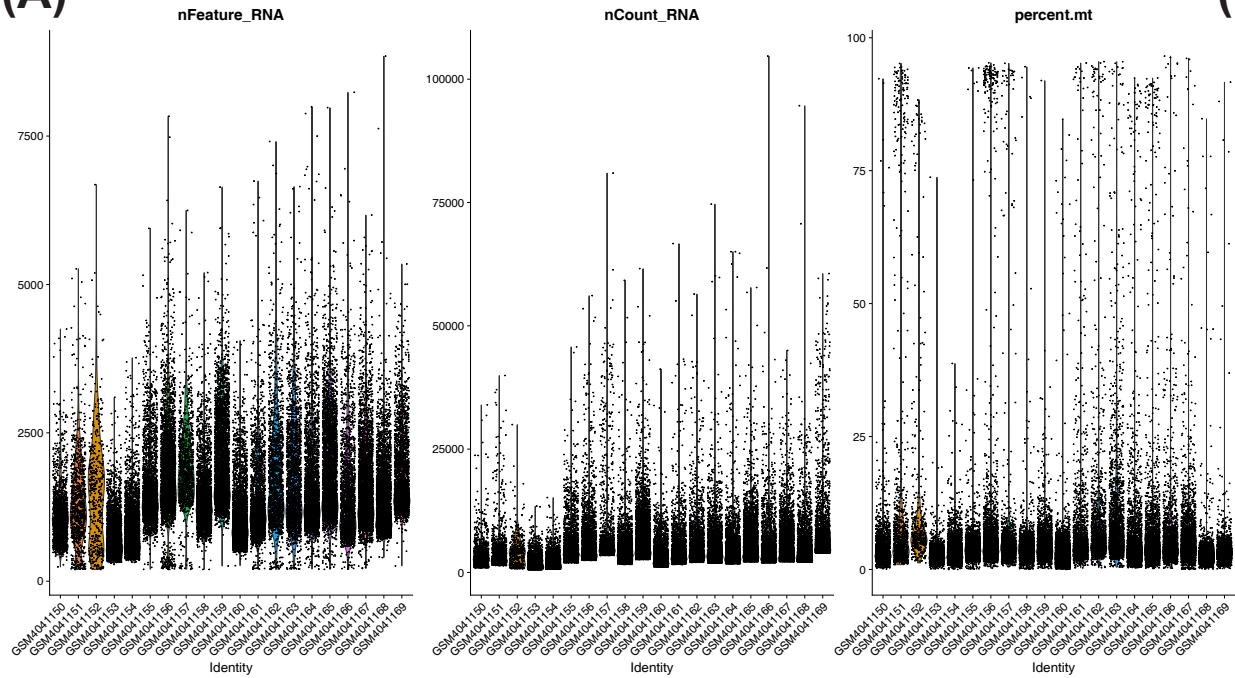**(B)**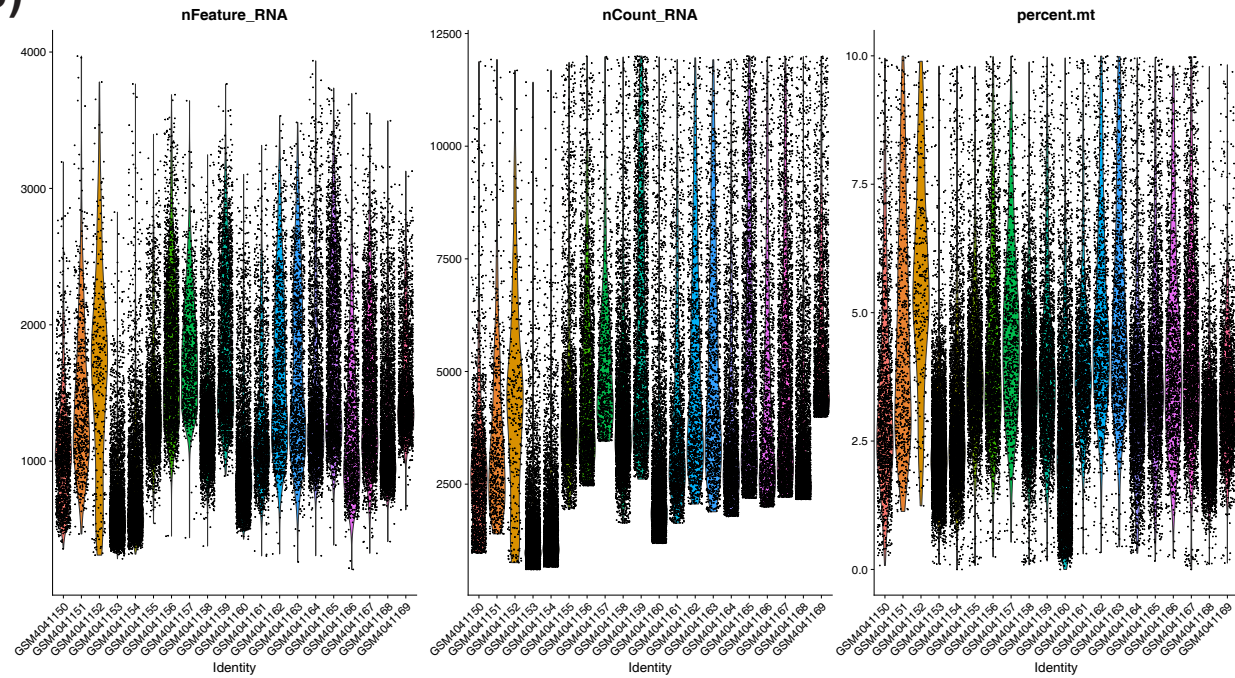**(C)**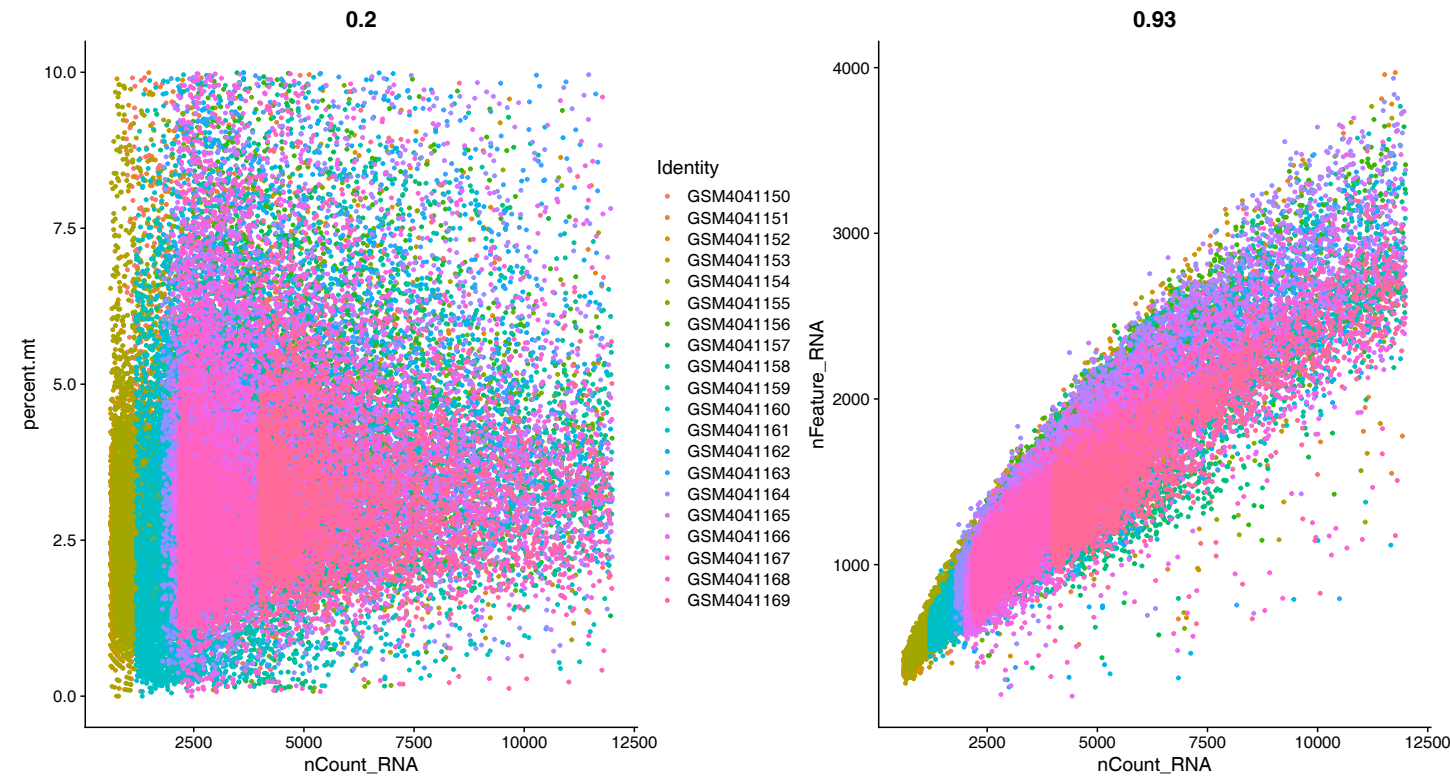**(D)**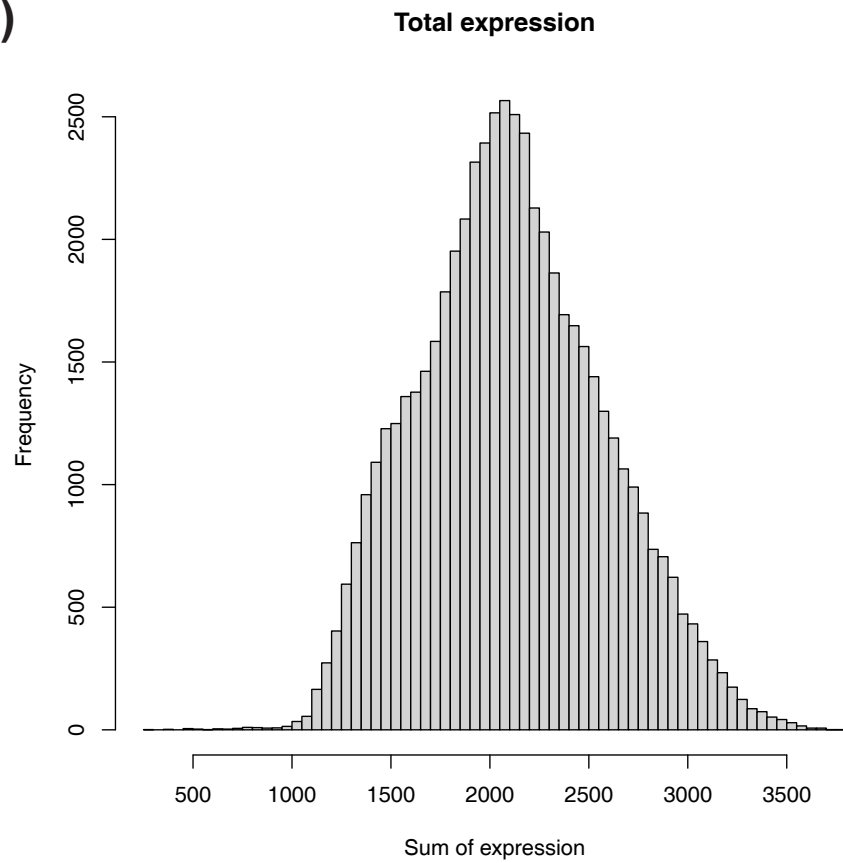**(E)**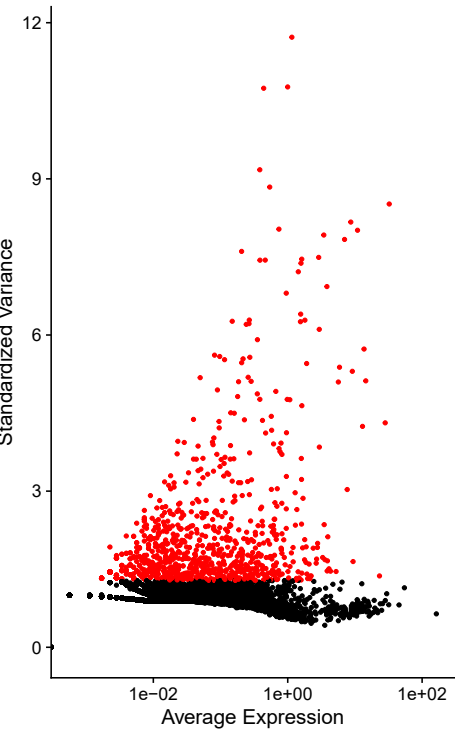**(F)**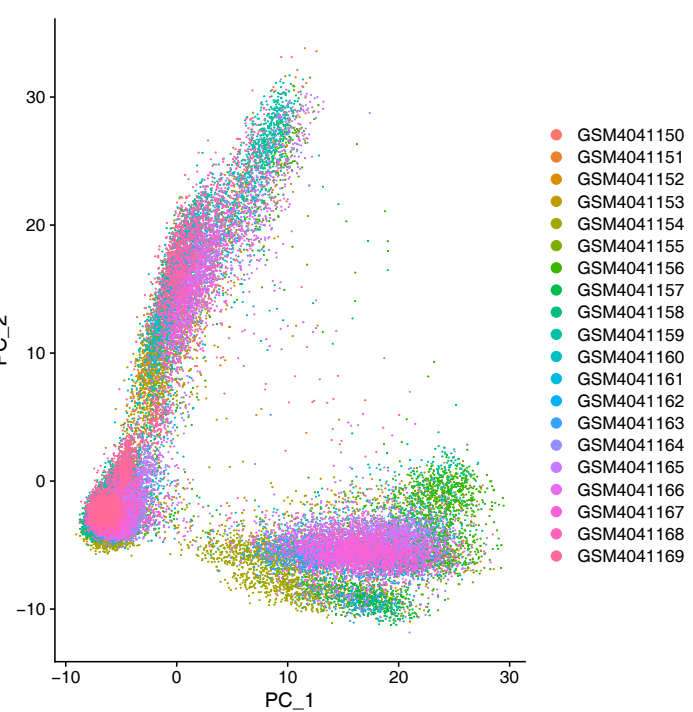**(G)**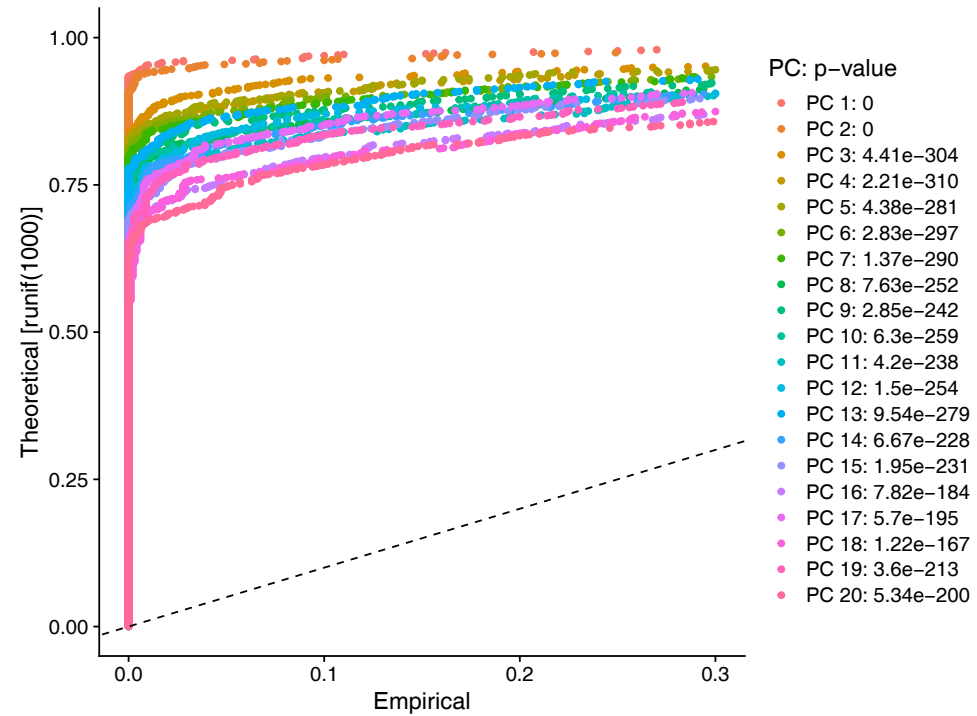**(H)**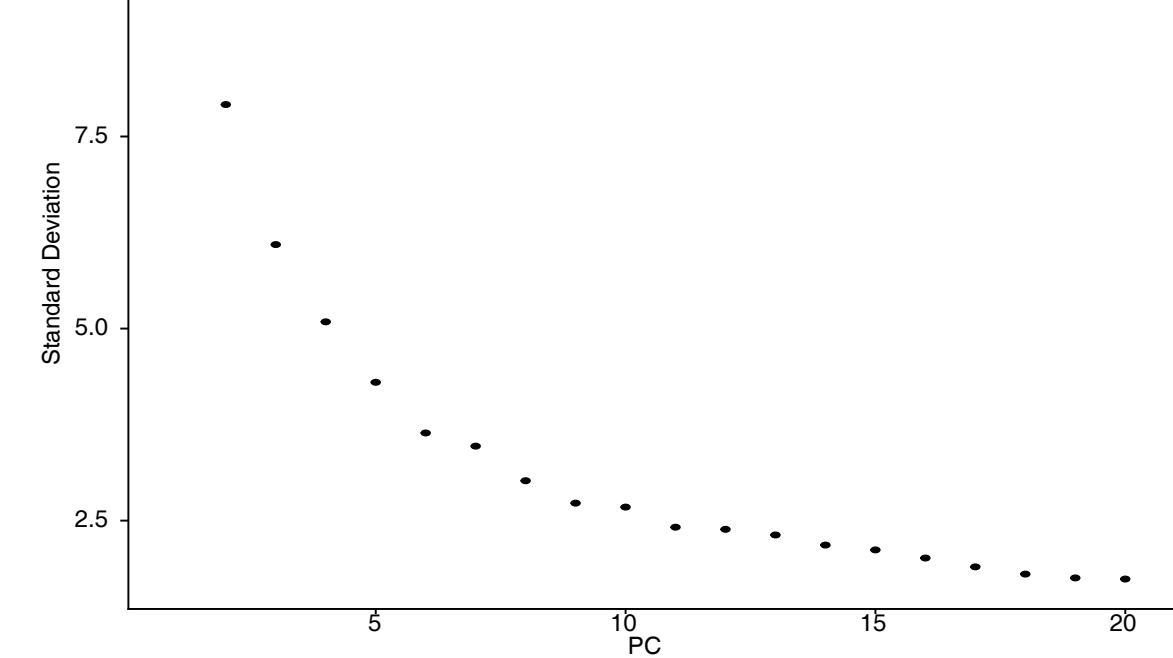**(I)**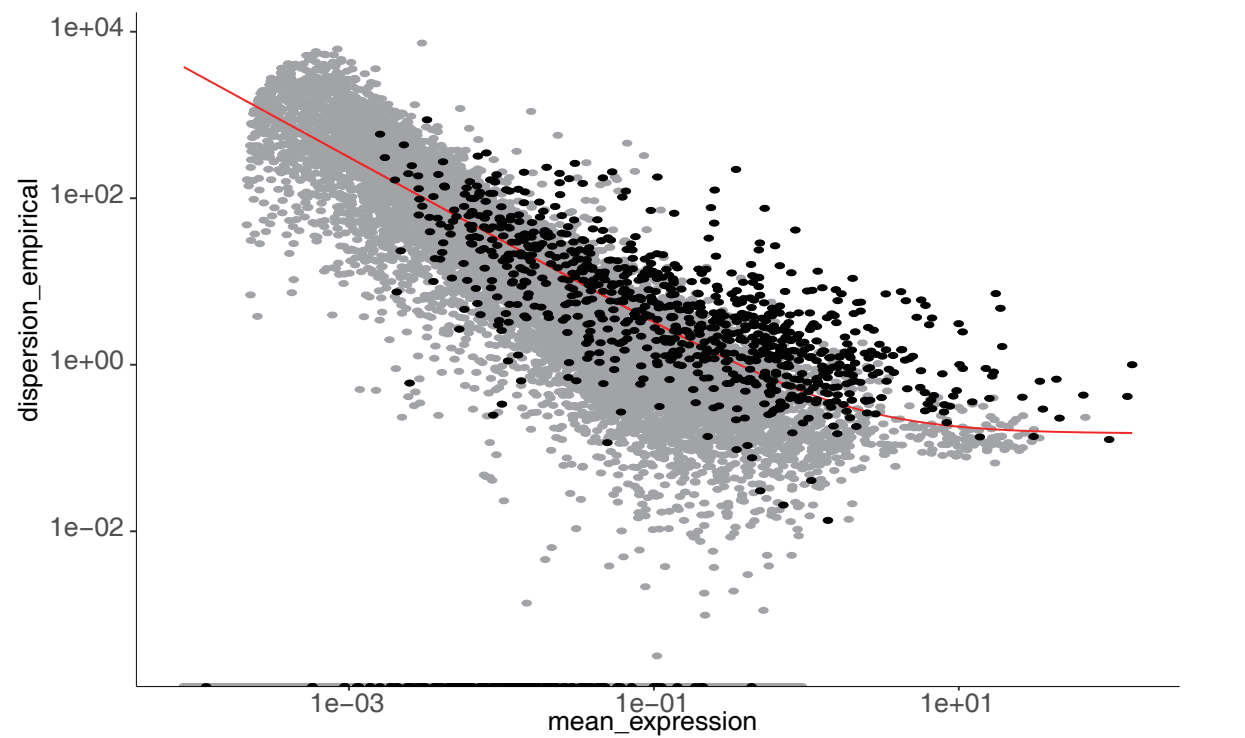

Supplement: Supplementary file 2 — Supporting Information 2 Figure S2. The quality control (QC) for single‐cell RNA‐seq analysis. (A) The nFeature, nCount, and mitochondria proportion detection before QC. (B) The nFeature, nCount, and mitochondria proportion detection after QC. (C) Outlier detection in samples. (D) The gene distribution frequency after standardization. (E) The volcano plot for Top 1000 HVG. (F) PCA analysis of cell clusters. (G) Regression analysis for the optimal number of clusters. (H) Scree plot for dimensionality reduction clustering. (I) The identifying HVGs of macrophages. [file GRP-2026-9985154-s001.pdf]
